# Modular Inference of Linear Types for Multiplicity-Annotated Arrows

**DOI:** 10.1007/978-3-030-44914-8_17

**Published:** 2020-04-18

**Authors:** Kazutaka Matsuda

**Affiliations:** ETH Zurich, Zurich, Switzerland; Graduate School of Information Sciences, Tohoku University, Sendai, 980-8579 Japan

**Keywords:** Linear Types, Type Inference, Qualified Typing.

## Abstract

Bernardy et al. [2018] proposed a linear type system $\lambda ^q_\rightarrow$ as a core type system of Linear Haskell. In the system, linearity is represented by annotated arrow types $A \rightarrow _m B$, where *m* denotes the multiplicity of the argument. Thanks to this representation, existing non-linear code typechecks as it is, and newly written linear code can be used with existing non-linear code in many cases. However, little is known about the type inference of $\lambda ^q_\rightarrow$. Although the Linear Haskell implementation is equipped with type inference, its algorithm has not been formalized, and the implementation often fails to infer principal types, especially for higher-order functions. In this paper, based on OutsideIn(X) [Vytiniotis et al., 2011], we propose an inference system for a rank 1 qualified-typed variant of $\lambda ^q_\rightarrow$, which infers principal types. A technical challenge in this new setting is to deal with ambiguous types inferred by naive qualified typing. We address this ambiguity issue through quantifier elimination and demonstrate the effectiveness of the approach with examples.

### 1 Introduction

Linearity is a fundamental concept in computation and has many applications. For example, if a variable is known to be used only once, it can be freely inlined without any performance regression [29]. In a similar manner, destructive updates are safe for such values without the risk of breaking referential transparency [32]. Moreover, linearity is useful for writing transformation on data that cannot be copied or discarded for various reasons, including reversible computation [19, 35] and quantum computation [2, 25]. Another interesting application of linearity is that it helps to bound the complexity of programs [1, 5, 13]

Linear type systems use types to enforce linearity. One way to design a linear type system is based on Curry-Howard isomorphism to linear logic. For example, in Wadler [33]'s type system, functions are linear in the sense that their arguments are used exactly once, and any exception to this must be marked by the type operator (!). Such an approach is theoretically elegant but cumbersome in programming; a program usually contains both linear and unrestricted code, and many manipulations concerning (!) are required in the latter and around the interface between the two. Thus, there have been several proposed approaches for more practical linear type systems [7, 21, 24, 28].

Among these approaches, a system called $\lambda^q_{\rightarrow}$, the core type system of Linear Haskell, stands out for its ability to have linear code in large unrestricted code bases [7]. With it, existing unrestricted code in Haskell typechecks in Linear Haskell without modification, and if one desires, some of the unrestricted code can be replaced with linear code, again without any special programming effort. For example, one can use the function *append* in an unrestricted context as $\lambda x.tail\ (append\ x\ x)$, regardless of whether *append* is a linear or unrestricted function. This is made possible by their representation of linearity. Specifically, they annotate function type with its argument's multiplicity ("linearity via arrows" [7]) as $A \rightarrow_m B$, where $m = 1$ means that the function of the type uses its argument linearly, and $m = \omega$ means that there is no restriction in the use of the argument, which includes all non-linear standard Haskell code. In this system, linear functions can be used in an unrestricted context if their arguments are unrestricted. Thus, there is no problem in using $append : \mathsf{List}\ A \rightarrow_1 \mathsf{List}\ A \rightarrow_1 \mathsf{List}\ A$ as above, provided that $x$ is unrestricted. This promotion of linear expressions to unrestricted ones is difficult in other approaches [21, 24, 28] (at least in the absence of bounded kind-polymorphism), where linearity is a property of a type (called "linearity via kinds" in [7]).

However, as far as we are aware, little is known about *type inference* for $\lambda^q_{\rightarrow}$. It is true that Linear Haskell is implemented as a fork[1] of the Glasgow Haskell Compiler (GHC), which of course comes with type inference. However, the algorithm has not been formalized and has limitations due to a lack of proper handling of multiplicity constraints. Indeed, Linear Haskell gives up handling complex constraints on multiplicities such as those with multiplications $p \cdot q$; as a result, Linear Haskell sometimes fails to infer principal types, especially for higher-order functions.[2] This limits the reusability of code. For example, Linear Haskell cannot infer an appropriate type for function composition to allow it to compose both linear and unrestricted functions.

A classical approach to have both separated constraint solving that works well with the usual unification-based typing and principal typing (for a rank 1 fragment) is qualified typing [15]. In qualified typing, constraints on multiplicities are collected, and then a type is qualified with it to obtain a principal type. Complex multiplicities are not a problem in unification as they are handled by a constraint solver. For example, consider $app = \lambda f.\lambda x.f\ x$. Suppose that $f$ has type $a \rightarrow_p b$, and $x$ has type $a$ (here we focus only on multiplicities). Let us write the multiplicities of $f$ and $x$ as $p_f$ and $p_x$, respectively. Since $x$ is passed to $f$, there is a constraint that the multiplicity $p_x$ of $x$ must be $\omega$ if the multiplicity $p$ of the $f$'s argument also is. In other words, $p_x$ must be no less than $p$, which is represented by inequality $p \leq p_x$ under the ordering $1 \leq \omega$. (We could represent the constraint as an equality $p_x = p \cdot p_x$, but using inequality is simpler here.)

[1] https://github.com/tweag/ghc/tree/linear-types

[2] Confirmed for commit `1c80dcb424e1401f32bf7436290dd698c739d906` at May 14, 2019.

For the multiplicity $p_f$ of $f$, there is no restriction because $f$ is used exactly once; linear use is always legitimate even when $p_f = \omega$. As a result, we obtain the inferred type $\forall p\, p_f\, p_x\, a\, b.\, p \le p_x \Rightarrow (a \to_p b) \to_{p_f} a \to_{p_x} b$ for *app*. This type is a principal one; it is intuitively because only the constraints that are needed for typing $\lambda f.\lambda x.f\ x$ are gathered. Having separate constraint solving phases itself is rather common in the context of linear typing [3, 4, 11, 12, 14, 23, 24, 29, 34]. Qualified typing makes the constraint solving phase local and gives the principal typing property that makes typing modular. In particular, in the context of linearity via kinds, qualified typing is proven to be effective [11, 24].

As qualified typing is useful in the context of linearity via kinds, one may expect that it also works well for linearity via arrows such as $\lambda^q_\to$. However, naive qualified typing turns out to be impractical for $\lambda^q_\to$ because it tends to infer ambiguous types [15, 27]. As a demonstration, consider a slightly different version of *app* defined as $app' = \lambda f.\lambda x.app\ f\ x$. Standard qualified typing [15, 31] infers the type

$$\forall q\, q_f\, q_x\, p_f\, p_x\, a\, b.\, (q \le q_x \wedge q_f \le p_f \wedge q_x \le p_x) \Rightarrow (a \to_q b) \to_{p_f} a \to_{p_x} b$$

by the following steps:

- The polymorphic type of *app* is instantiated to $(a \to_q b) \to_{q_f} a \to_{q_x} b$ and yields a constraint $q \le q_x$ (again we focus only on multiplicity constraints).
- Since $f$ is used as the first argument of *app*, $f$ must have type $a \to_q b$. Also, since the multiplicity of *app*'s first argument is $q_f$, there is a restriction on the multiplicity of $f$, say $p_f$, that $q_f \le p_f$.
- Similarly, since $x$ is used as the second argument of *app*, $x$ must have type $a$, and there is a constraint on the multiplicity of $x$, say $p_x$, that $q_x \le p_x$.

This inference is unsatisfactory, as the inferred type leaks internal details and is ambiguous [15, 27] in the sense that one cannot determine $q_f$ and $q_x$ from an instantiation of $(a \to_q b) \to_{p_f} a \to_{p_x} b$. Due to this ambiguity, the types of *app* and *app*' are not judged as equivalent; in fact, the standard qualified typing algorithms [15, 31] reject $app' : \forall p\, p_f\, p_x\, a\, b.\, p \le p_x \Rightarrow (a \to_p b) \to_{p_f} a \to_{p_x} b$. We conjecture that the issue of inferring ambiguous types is intrinsic to linearity via arrows because of the separation of multiplicities and types, unlike the case of linearity via kinds, where multiplicities are always associated with types. Simple solutions such as rejecting ambiguous types are not desirable as this case appears very often. Defaulting ambiguous variables (such as $q_f$ and $q_x$) to 1 or $\omega$ is not a solution either because it loses principality in general.

In this paper, we propose a type inference method for a rank 1 qualified-typed variant of $\lambda^q_\to$, in which the ambiguity issue is addressed without compromising principality. Our type inference system is built on top of OutsideIn(X) [31], an inference system for qualified types used in GHC, which can handle local assumptions to support **let**, existential types, and GADTs. An advantage of using OutsideIn(X) is that it is parameterized over theory X of constraints. Thus, applying it to linear typing boils down to choosing an appropriate X. We choose X carefully so that the representation of constraints is closed under quantifier elimination, which is the key to addressing the ambiguity issue. Specifically, in this paper:

– We present a qualified typing variant of a rank-1 fragment of $\lambda^q_\to$ without local definitions, in which manipulation of multiplicities is separated from the standard unification-based typing (Sect. 2).
– We give an inference method for the system based on gathering constraints and solving them afterward (Sect. 3). This step is mostly standard, except that we solve multiplicity constraints in time polynomial in their sizes.
– We address the ambiguity issue by quantifier elimination under the assumption that multiplicities do not affect runtime behavior (Sect. 4).
– We extend our technique to local assumptions (Sect. 5), which enables **let** and GADTs, by showing that the disambiguation in Sect. 4 is compatible with OutsideIn(X).
– We report experimental results using our proof-of-concept implementation (Sect. 6). The experiments show that the system can infer unambiguous principal types for selected functions from Haskell's `Prelude`, and performs well with acceptable overhead.

Finally, we discuss related work (Sect. 7) and then conclude the paper (Sect. 8). The prototype implementation is available as a part of a reversible programming system Sparcl, available from https://bitbucket.org/kztk/partially-reversible-lang-impl/. Due to space limitation, we omit some proofs from this paper, which can be found in the full version [20].

### 2 Qualified-Typed Variant of $\lambda^q_\to$

In this section, we introduce a qualified-typed [15] variant of $\lambda^q_\to$ [7] for its rank 1 fragment, on which we base our type inference. Notable differences to the original $\lambda^q_\to$ include: (1) multiplicity abstractions and multiplicity applications are implicit (as type abstractions and type applications), (2) this variant uses qualified typing [15], (3) conditions on multiplicities are inequality based [6], which gives better handling of multiplicity variables, and (4) local definitions are excluded as we postpone the discussions to Sect. 5 due to their issues in the handling of local assumptions in qualified typing [31].

#### 2.1 Syntax of Programs

Programs and expressions, which will be typechecked, are given below.

$$\begin{array}{lcl} \mathit{prog} & ::= & \mathit{bind}_1; \dots ; \mathit{bind}_n \\ \mathit{bind} & ::= & f = e \mid f : A = e \\ e & ::= & x \mid \lambda x.e \mid e_1\ e_2 \mid \mathsf{C}\ \overline{e} \mid \mathbf{case}\ e_0\ \mathbf{of}\ \{\mathsf{C}_i\ \overline{x_i} \to e_i\}_i \end{array}$$

A program is a sequence of bindings with or without type annotations, where bound variables can appear in following bindings. As mentioned at the beginning

| | | | | | |
|---|---|---|---|---|---|
| $A, B$ | $::= \forall \overline{pa}.Q \Rightarrow \tau$ | (polytypes) | $Q$ | $::= \bigwedge_i \phi_i$ | (constraints) |
| $\sigma, \tau$ | $::= a \mid \mathsf{D}\,\overline{\mu}\,\overline{\tau} \mid \sigma \to_\mu \tau$ | (monotypes) | $\phi$ | $::= M \leq M'$ | (predicates) |
| $\mu$ | $::= p \mid 1 \mid \omega$ | (multiplicities) | $M, N$ | $::= \prod_i \mu_i$ | (multiplications) |

**Fig. 1.** Types and related notions: $a$ and $p$ are type and multiplicity variables, respectively, and $\mathsf{D}$ represents a type constructor.

of this section, we shall postpone the discussions of local bindings (i.e., **let**) to Sect. 5. Expressions consist of variables $x$, applications $e_1\ e_2$, $\lambda$-abstractions $\lambda x.e$, constructor applications $\mathsf{C}\ \overline{e}$, and (shallow) pattern matching **case** $e_0$ **of** $\{\mathsf{C}_i\ \overline{x_i} \to e_i\}_i$. For simplicity, we assume that constructors are fully-applied and patterns are shallow. As usual, patterns $\mathsf{C}_i\ \overline{x_i}$ must be linear in the sense that each variable in $\overline{x_i}$ is different. Programs are assumed to be appropriately $\alpha$-renamed so that newly introduced variables by $\lambda$ and patterns are always fresh. We do not require the patterns of a **case** expression to be exhaustive or no overlapping, following the original $\lambda^q_\to$ [7]; the linearity in $\lambda^q_\to$ cares only for successful computations. Unlike the original $\lambda^q_\to$, we do not annotate $\lambda$ and **case** with the multiplicity of the argument and the scrutinee, respectively.

Constructors play an important role in $\lambda^q_\to$. As we will see later, they can be used to witness unrestrictedness, similarly to ! of !$e$ in a linear type system [33].

### 2.2 Types

Types and related notations are defined in Fig. 1. Types are separated into monotypes and polytypes (or, type schemes). Monotypes consist of (rigid) type variables $a$, datatypes $\mathsf{D}\ \overline{\mu}\ \overline{\tau}$, and multiplicity-annotated function types $\tau_1 \to_\mu \tau_2$. Here, a multiplicity $\mu$ is either 1 (linear), $\omega$ (unrestricted), or a (rigid) multiplicity variable $p$. Polytypes have the form $\forall \overline{pa}.Q \Rightarrow \tau$, where $Q$ is a constraint that is a conjunction of predicates. A predicate $\phi$ has the form of $M \leq M'$, where $M'$ and $M$ are multiplications of multiplicities. We shall sometimes treat $Q$ as a set of predicates, which means that we shall rewrite $Q$ according to contexts by the idempotent commutative monoid laws of $\wedge$. We call both multiplicity ($p$) and type ($a$) variables *type-level variables*, and write $\mathsf{ftv}(\overline{t})$ for the set of free type-level variables in syntactic objects (such as types and constraints) $\overline{t}$.

The relation ($\leq$) and operator ($\cdot$) in predicates denote the corresponding relation and operator on $\{1, \omega\}$, respectively. On $\{1, \omega\}$, ($\leq$) is defined as the reflexive closure of $1 \leq \omega$; note that $(\{1, \omega\}, \leq)$ forms a total order. Multiplication ($\cdot$) on $\{1, \omega\}$ is defined by

$$1 \cdot m = m \cdot 1 = m \qquad \omega \cdot m = m \cdot \omega = \omega.$$

For simplicity, we shall sometimes omit ($\cdot$) and write $m_1 m_2$ for $m_1 \cdot m_2$. Note that, for $m_1, m_2 \in \{1, \omega\}$, $m_1 \cdot m_2$ is the least upper bound of $m_1$ and $m_2$ with respect to $\leq$. As a result, $m_1 \cdot m_2 \leq m$ holds if and only if $(m_1 \leq m) \wedge (m_2 \leq m)$ holds; we will use this property for efficient handling of constraints (Sect. 3.2).

We assume a fixed set of constructors given beforehand. Each constructor is assigned a type of the form $\forall \overline{pa}.\ \tau_1 \to_{\mu_1} \dots \to_{\mu_{n_1}} \tau_n \to_{\mu_n} \mathsf{D}\ \overline{p}\ \overline{a}$ where each $\tau_i$ and $\mu_i$ do not contain free type-level variables other than $\{\overline{pa}\}$, i.e., $\bigcup_i \mathsf{ftv}(\tau_i, \mu_i) \subseteq \{\overline{pa}\}$. For simplicity, we write the above type as $\forall \overline{pa}.\ \overline{\tau} \to_{\overline{\mu}} \mathsf{D}\ \overline{p}\ \overline{a}$. We assume that types are well-kinded, which effectively means that $\mathsf{D}$ is applied to the same numbers of multiplicity arguments and type arguments among the constructor types. Usually, it suffices to use constructors of linear function types as below because they can be used in both linear and unrestricted code.

$$(-,-) : \forall a\, b.\ a \to_1 b \to_1 a \otimes b$$
$$\mathsf{Nil} : \forall a.\ \mathsf{List}\ a \qquad \mathsf{Cons} : \forall a.\ a \to_1 \mathsf{List}\ a \to_1 \mathsf{List}\ a$$

In general, constructors can encapsulate arguments' multiplicities as below, which is useful when a function returns both linear and unrestricted results.

$$\mathsf{MkUn} : \forall a.\ a \to_\omega \mathsf{Un}\ a \qquad \mathsf{MkMany} : \forall p\, a.\ a \to_p \mathsf{Many}\ p\ a$$

For example, a function that reads a value from a mutable array at a given index can be given as a primitive of type $\mathit{readMArray} : \forall a.\ \mathsf{MArray}\ a \to_1 \mathsf{Int} \to_\omega (\mathsf{MArray}\ a \otimes \mathsf{Un}\ a)$ [7]. Multiplicity-parameterized constructors become useful when the multiplicity of contents can vary. For example, the type $\mathsf{IO_L}\ p\ a$ with the constructor $\mathsf{MkIO_L} : (\mathsf{World} \to_1 (\mathsf{World} \otimes \mathsf{Many}\ p\ a)) \to_1 \mathsf{IO_L}\ p\ a$ can represent the IO monad [7] with methods $\mathit{return} : \forall p\, a.\ a \to_p \mathsf{IO_L}\ p\ a$ and $(\ggg\!=) : \forall p\, q\, a\, b.\ \mathsf{IO_L}\ p\ a \to_1 (a \to_p \mathsf{IO_L}\ q\ b) \to_1 \mathsf{IO_L}\ q\ b$.

### 2.3 Typing Rules

Our type system uses two sorts of environments A *typing environment* maps variables into polytypes (as usual in non-linear calculi), and a *multiplicity environment* maps variables into multiplications of multiplicities. This separation of the two will be convenient when we discuss type inference. As usual, we write $x_1 : A_1, \dots, x_n : A_n$ instead of $\{x_1 \mapsto A_1, \dots, x_n \mapsto A_n\}$ for typing environments. For multiplicity environments, we use multiset-like notation as $x_1{}^{M_1}, \dots, x_n{}^{M_n}$.

We use the following operations on multiplicity environments:[3]

$$(\Delta_1 + \Delta_2)(x) = \begin{cases} \omega & \text{if } x \in \mathsf{dom}(\Delta_1) \cap \mathsf{dom}(\Delta_2) \\ \Delta_i(x) & \text{if } x \in \mathsf{dom}(\Delta_i) \setminus \mathsf{dom}(\Delta_j)\ (i \neq j \in \{1,2\}) \end{cases}$$
$$(\mu\Delta)(x) = \mu \cdot \Delta(x)$$
$$(\Delta_1 \sqcup \Delta_2)(x) = \begin{cases} \Delta_1(x) \cdot \Delta_2(x) & \text{if } x \in \mathsf{dom}(\Delta_1) \cap \mathsf{dom}(\Delta_2) \\ \omega & \text{if } x \in \mathsf{dom}(\Delta_i) \setminus \mathsf{dom}(\Delta_j)\ (i \neq j \in \{1,2\}) \end{cases}$$

[3] In these definitions, we implicitly consider multiplicity 0 and regard $\Delta(x) = 0$ if $x \notin \mathsf{dom}(\Delta)$. It is natural that $0 + m = m + 0$. With 0, multiplication $\cdot$, which is extended as $0 \cdot m = m \cdot 0 = 0$, no longer computes the least upper bound. Therefore, we use $\sqcup$ for the last definition; in fact, the definition corresponds to the pointwise computation of $\Delta_1(x) \sqcup \Delta_2(x)$, where $\leq$ is extended as $0 \leq \omega$ but not $0 \leq 1$. This treatment of 0 coincides with that in the Linear Haskell proposal [26].

$$\frac{Q;\varGamma;\varDelta' \vdash e : \tau' \quad Q \models \varDelta = \varDelta' \quad Q \models \tau \sim \tau'}{Q;\varGamma;\varDelta \vdash e : \tau}\,\textsc{Eq} \qquad \frac{\varGamma(x) = \forall \overline{pa}.Q' \Rightarrow \tau \quad Q \models Q'[\overline{p \mapsto \mu}] \quad Q \models x^1 \leq \varDelta}{Q;\varGamma;\varDelta \vdash x : \tau[\overline{p \mapsto \mu}, \overline{a \mapsto \tau}]}\,\textsc{Var}$$

$$\frac{Q;\varGamma, x : \sigma;\varDelta, x^{\mu} \vdash e : \tau}{Q;\varGamma;\varDelta \vdash \lambda x.e : \sigma \to_{\mu} \tau}\,\textsc{Abs} \qquad \frac{Q;\varGamma;\varDelta_1 \vdash e_1 : \sigma \to_{\mu} \tau \quad Q;\varGamma;\varDelta_2 \vdash e_2 : \sigma}{Q;\varGamma;\varDelta_1 + \mu\varDelta_2 \vdash e_1\ e_2 : \tau}\,\textsc{App}$$

$$\frac{\mathsf{C} : \forall \overline{pa}.\,\overline{\tau} \to_{\overline{\nu}} \mathsf{D}\ \overline{p}\ \overline{a} \quad \{Q;\varGamma;\varDelta_i \vdash e_i : \tau_i[\overline{p \mapsto \mu}, \overline{a \mapsto \sigma}]\}_i}{Q;\varGamma;\omega\varDelta_0 + \sum_i \nu_i[\overline{p \mapsto \mu}]\varDelta_i \vdash \mathsf{C}\ \overline{e} : \mathsf{D}\ \overline{\mu}\ \overline{\sigma}}\,\textsc{Con}$$

$$\frac{Q;\varGamma;\varDelta_0 \vdash e_0 : \mathsf{D}\ \overline{\mu}\ \overline{\sigma} \quad \left\{\begin{matrix}\mathsf{C}_i : \forall \overline{pa}.\,\overline{\tau_i} \to_{\overline{\nu_i}} \mathsf{D}\ \overline{p}\ \overline{a} \\ Q;\varGamma, \overline{x_i : \tau_i[\overline{p \mapsto \mu}, \overline{a \mapsto \sigma}]};\varDelta_i, \overline{{x_i}^{\mu_0 \nu_i[\overline{p \mapsto \mu}]}} \vdash e_i : \tau'\end{matrix}\right\}_i}{Q;\varGamma;\mu_0\varDelta_0 + \bigsqcup_i \varDelta_i \vdash \textbf{case}\ e_0\ \textbf{of}\ \{\mathsf{C}_i\ \overline{x_i} \to e_i\}_i : \tau'}\,\textsc{Case}$$

**Fig. 2.** Typing relation for expressions

Intuitively, $\varDelta(x)$ represents the number of uses of $x$. So, in the definition of $\varDelta_1 + \varDelta_2$, we have $(\varDelta_1 + \varDelta_2)(x) = \omega$ if $x \in \mathsf{dom}(\varDelta_1) \cap \mathsf{dom}(\varDelta_2)$ because this condition means that $x$ is used in two places. Operation $\varDelta_1 \sqcup \varDelta_2$ is used for **case** branches. Suppose that a branch $e_1$ uses variables as $\varDelta_1$ and another branch $e_2$ uses variables as $\varDelta_2$. Then, putting the branches together, variables are used as $\varDelta_1 \sqcup \varDelta_2$. The definition says that $x$ is considered to be used linearly in the two branches put together if and only if both branches use $x$ linearly, where non-linear use includes unrestricted use ($\varDelta_i(x) = \omega$) and non-use ($x \notin \mathsf{dom}(\varDelta)$).

We write $Q \models Q'$ if $Q$ logically entails $Q'$. That is, for any valuation of multiplicity variables $\theta(p) \in \{1, \omega\}$, $Q'\theta$ holds if $Q\theta$ does. For example, we have $p \leq r \wedge r \leq q \models p \leq q$. We extend the notation to multiplicity environments and write $Q \models \varDelta_1 \leq \varDelta_2$ if $\mathsf{dom}(\varDelta_1) \subseteq \mathsf{dom}(\varDelta_2)$ and $Q \models \bigwedge_{x \in \mathsf{dom}(\varDelta)} \varDelta_1(x) \leq \varDelta_2(x) \wedge \bigwedge_{x \in \mathsf{dom}(\varDelta_2) \setminus \mathsf{dom}(\varDelta_1)} \omega \leq \varDelta_2(x)$ hold. We also write $Q \models \varDelta_1 = \varDelta_2$ if both $Q \models \varDelta_1 \leq \varDelta_2$ and $Q \models \varDelta_2 \leq \varDelta_1$ hold. We then have the following properties.

**Lemma 1.** Suppose $Q \models \varDelta \leq \varDelta'$ and $Q \models \varDelta = \varDelta_1 + \varDelta_2$. Then, there are some $\varDelta'_1$ and $\varDelta'_2$ such that $Q \models \varDelta' = \varDelta'_1 + \varDelta'_2$, $Q \models \varDelta_1 \leq \varDelta'_1$ and $Q \models \varDelta_2 \leq \varDelta'_2$. □

**Lemma 2.** $Q \models \mu\varDelta \leq \varDelta'$ implies $Q \models \varDelta \leq \varDelta'$. □

**Lemma 3.** $Q \models \varDelta_1 \sqcup \varDelta_2 \leq \varDelta'$ implies $Q \models \varDelta_1 \leq \varDelta'$ and $Q \models \varDelta_2 \leq \varDelta'$. □

Constraints $Q$ affect type equality; for example, under $Q = p \leq q \wedge q \leq p$, $\sigma \to_p \tau$ and $\sigma \to_q \tau$ become equivalent. Formally, we write $Q \models \tau \sim \tau'$ if $\tau\theta = \tau'\theta$ for any valuation $\theta$ of multiplicity variables that makes $Q\theta$ true.

Now, we are ready to define the *typing judgment for expressions*, $Q;\varGamma;\varDelta \vdash e : \tau$, which reads that under assumption $Q$, typing environment $\varGamma$, and multiplicity environment $\varDelta$, expression $e$ has monotype $\tau$, by the typing rules in Fig. 2. Here, we assume $\mathsf{dom}(\varDelta) \subseteq \mathsf{dom}(\varGamma)$. Having $x \in \mathsf{dom}(\varGamma) \setminus \mathsf{dom}(\varDelta)$ means that the multiplicity of $x$ is essentially 0 in $e$.

Rule Eq says that we can replace $\tau$ and $\varDelta$ with equivalent ones in typing.

$$\frac{}{\Gamma \vdash \varepsilon}\textsc{Empty} \qquad \frac{Q;\Gamma;\Delta \vdash e : \tau \quad \overline{pa} = \mathsf{ftv}(Q,\tau) \quad \Gamma, f : \forall \overline{pa}.Q \Rightarrow \tau \vdash \mathit{prog}}{\Gamma \vdash f = e; \mathit{prog}}\textsc{Bind}$$

$$\frac{Q;\Gamma;\Delta \vdash e : \tau \quad \overline{pa} = \mathsf{ftv}(Q,\tau) \quad \Gamma, f : \forall \overline{pa}.Q \Rightarrow \tau \vdash \mathit{prog}}{\Gamma \vdash f : (\forall \overline{pa}.Q \Rightarrow \tau) = e; \mathit{prog}}\textsc{BindA}$$

**Fig. 3.** Typing rules for programs

Rule Var says that $x$ is used once in a variable expression $x$, but it is safe to regard that the expression uses $x$ more than once and uses other variables $\omega$ times. At the same time, the type $\forall \overline{pa}.Q' \Rightarrow \tau$ of $x$ instantiated to $\tau[\overline{p \mapsto \mu}, \overline{a \mapsto \sigma}]$ with yielding constraints $Q'[\overline{p \mapsto \mu}]$, which must be entailed from $Q$.

Rule Abs says that $\lambda x.e$ has type $\sigma \to_\mu \tau$ if $e$ has type $\tau$, assuming that the use of $x$ in $e$ is $\mu$. Unlike the original $\lambda^q_{\to}$ [7], in our system, multiplicity annotations on arrows must be $\mu$, i.e., 1, $\omega$, or a multiplicity variable, instead of $M$. This does not limit the expressiveness because such general arrow types can be represented by type $\sigma \to_p \tau$ with constraints $p \leq M \wedge M \leq p$.

Rule App sketches an important principle in $\lambda^q_{\to}$; when an expression with variable use $\Delta$ is used $\mu$-many times, the variable use in the expression becomes $\mu\Delta$. Thus, since we pass $e_2$ (with variable use $\Delta_2$) to $e_1$, where $e_1$ uses the argument $\mu$-many times as described in its type $\sigma \to_\mu \tau$, the use of variables in $e_2$ of $e_1\ e_2$ becomes $\mu\Delta_2$. For example, for $(\lambda y.42)\ x$, $x$ is considered to be used $\omega$ times because $(\lambda y.42)$ has type $\sigma \to_\omega \mathsf{Int}$ for any $\sigma$.

Rule Con is nothing but a combination of Var and App. The $\omega\Delta_0$ part is only useful when $\mathsf{C}$ is nullary; otherwise, we can weaken $\Delta$ at leaves.

Rule Case is the most complicated rule in this type system. In this rule, $\mu_0$ represents how many times the scrutinee $e_0$ is used in the **case**. If $\mu_0 = \omega$, the pattern bound variables can be used unrestrictedly, and if $\mu_0 = 1$, the pattern bound variables can be used according to the multiplicities of the arguments of the constructor.[4] Thus, in the $i$th branch, variables in $\overline{x_i}$ can be used as $\overline{\mu_0\nu_i[\overline{p \mapsto \mu}]}$, where $\mu_i[\overline{p \mapsto \mu}]$ represents the multiplicities of the arguments of the constructor $\mathsf{C}_i$. Other than $\overline{x_i}$, each branch body $e_i$ can contain free variables used as $\Delta_i$. Thus, the uses of free variables in the whole branch bodies are summarized as $\bigsqcup_i \Delta_i$. Recall that the **case** uses the scrutinee $\mu_0$ times; thus, the whole uses of variables are estimated as $\mu_0\Delta_0 + \bigsqcup_i \Delta_i$.

Then, we define the *typing judgment for programs*, $\Gamma \vdash \mathit{prog}$, which reads that program *prog* is well-typed under $\Gamma$, by the typing rules in Fig. 3. At this place, the rules Bind and BindA have no significant differences; their difference will be clear when we discuss type inference. In the rules Bind and BindA, we assumed that $\Gamma$ contains no free type-level variables. Therefore, we can safely generalize all free type-level variables in $Q$ and $\tau$. We do not check the use $\Delta$ in both rules

[4] This behavior, inherited from $\lambda^q_{\to}$ [7], implies the isomorphism $!(A \otimes B) \equiv\ !A \otimes !B$, which is not a theorem in the standard linear logic. The isomorphism intuitively means that unrestricted products can (only) be constructed from unrestricted components, as commonly adopted in linearity-via-kind approaches [11,21,24,28,29].

as bound variables are assumed to be used arbitrarily many times in the rest of the program; that is, the multiplicity of a bound variable is $\omega$ and its body uses variable as $\omega\Delta$, which maps $x \in \mathsf{dom}(\Delta)$ to $\omega$ and has no free type-level variables.

#### 2.4 Metatheories

Lemma 4 is the standard weakening property. Lemma 5 says that we can replace $Q$ with a stronger one, Lemma 6 says that we can replace $\Delta$ with a greater one, and Lemma 7 says that we can substitute type-level variables in a term-in-context without violating typeability. These lemmas state some sort of weakening, and the last three lemmas clarify the goal of our inference system discussed in Sect. 3.

**Lemma 4.** $Q; \Gamma; \Delta \vdash e : \tau$ implies $Q; \Gamma, x : A; \Delta \vdash e : \tau$. □

**Lemma 5.** $Q; \Gamma; \Delta \vdash e : \tau$ and $Q' \models Q$ implies $Q'; \Gamma; \Delta \vdash e : \tau$. □

**Lemma 6.** $Q; \Gamma; \Delta \vdash e : \tau$ and $Q \models \Delta \leq \Delta'$ implies $Q; \Gamma; \Delta' \vdash e : \tau$. □

**Lemma 7.** $Q; \Gamma; \Delta \vdash e : \tau$ implies $Q\theta; \Gamma\theta; \Delta\theta \vdash e : \tau\theta$. □

We have the following form of the substitution lemma:

**Lemma 8 (Substitution).** Suppose $Q_0; \Gamma, \overline{x : \sigma}; \Delta_0, \overline{x^{\mu}} \vdash e : \tau$, and $Q_i; \Gamma; \Delta_i \vdash e'_i : \sigma_i$ for each $i$. Then, $Q_1 \wedge \bigwedge_i Q_i; \Gamma; \Delta_0 + \sum_i \mu_i \Delta_i \vdash e[\overline{x \mapsto e'}] : \tau$. □

*Subject Reduction* We show the subject reduction property for a simple call-by-name semantics. Consider the standard small-step call-by-name relation $e \longrightarrow e'$ with the following $\beta$-reduction rules (we omit the congruence rules):

$$(\lambda x.e_1)\ e_2 \longrightarrow e_1[x \mapsto e_2] \qquad \textbf{case}\ \mathsf{C}_j\ \overline{e_j}\ \textbf{of}\ \{\mathsf{C}_i\ \overline{x_i} \to e'_i\}_i \longrightarrow e'_j[\overline{x_j \mapsto e_j}]$$

Then, by Lemma 8, we have the following subjection reduction property:

**Lemma 9 (Subject Reduction).** $Q; \Gamma; \Delta \vdash e : \tau$ and $e \longrightarrow e'$ implies $Q; \Gamma; \Delta \vdash e' : \tau$. □

Lemma 9 holds even for the call-by-value reduction, though with a caveat. For a program $f_1 = e_1; \dots; f_n = e_n$, it can happen that some $e_i$ is typed only under unsatisfiable (i.e., conflicting) $Q_i$. As conflicting $Q_i$ means that $e_i$ is essentially ill-typed, evaluating $e_i$ may not be safe. However, the standard call-by-value strategy evaluates $e_i$, even when $f_i$ is not used at all and thus the type system does not reject this unsatisfiability. This issue can be addressed by the standard witness-passing transformation [15] that converts programs so that $Q \Rightarrow \tau$ becomes $W_Q \to \tau$, where $W_Q$ represents a set of witnesses of $Q$. Nevertheless, it would be reasonable to reject conflicting constraints locally.

We then state the correspondence with the original system [7] (assuming the modification [6] for the variable case[5]) to show that the qualified-typed version

[5] In the premise of Var, the original [7] uses $\exists \Delta'.\ \Delta = x^1 + \omega\Delta'$, which is modified to $x^1 \leq \Delta$ in [6]. The difference between the two becomes clear when $\Delta(x) = p$, for which the former one does not hold as we are not able to choose $\Delta'$ depending on $p$.

captures the linearity as the original. While the original system assumes the call-by-need evaluation, Lemma 9 could be lifted to that case.

**Theorem 1.** *If* $\top; \Gamma; \Delta \vdash e : \tau$ *where* $\Gamma$ *contains only monotypes,* $e$ *is also well-typed in the original* $\lambda^q_{\rightarrow}$ *under some environment.* □

The main reason for the monotype restriction is that our polytypes are strictly more expressive than their (rank-1) polytypes. This extra expressiveness comes from predicates of the form $\cdots \leq M \cdot M'$. Indeed, $f = \lambda x.\mathbf{case}\ x\ \mathbf{of}\ \{\mathsf{MkMany}\ y \to (y, y)\}$ has type $\forall p\, q\, a.\, \omega \leq p \cdot q \Rightarrow \mathsf{MkMany}\ p\ a \to_q a \otimes a$ in our system, while it has three incomparable types in the original $\lambda^q_{\rightarrow}$.

### 3 Type Inference

In this section, we give a type inference method for the type system in the previous section. Following [31, Section 3], we adopt the standard two-phase approach; we first gather constraints on types and then solve them. As mentioned in Sect. 1, the inference system described here has the issue of ambiguity, which will be addressed in Sect. 4.

#### 3.1 Inference Algorithm

We first extend types $\tau$ and multiplicities $\mu$ to include *unification variables.*

$$\tau ::= \cdots \mid \alpha \qquad \mu ::= \cdots \mid \pi$$

We call $\alpha/\pi$ a unification type/multiplicity variable, which will be substituted by a concrete type/multiplicity (including rigid variables) during the inference. Similarly to $\mathsf{ftv}(\bar{t})$, we write $\mathsf{fuv}(\bar{t})$ for the unification variables (of both sorts) in $\bar{t}$, where each $t_i$ ranges over any syntactic element (such as $\tau$, $Q$, $\Gamma$, and $\Delta$).

Besides $Q$, the algorithm will generate equality constraints $\tau \sim \tau'$. Formally, the sets of *generated constraints* $C$ and *generated predicates* $\psi$ are given by

$$C ::= \bigwedge_i \psi_i \qquad \psi ::= \phi \mid \tau \sim \tau'$$

Then, we define *type inference judgment for expressions*, $\Gamma \blacktriangleright e : \tau \rightsquigarrow \Delta; C$, which reads that, given $\Gamma$ and $e$, type $\tau$ is inferred together with variable use $\Delta$ and constraints $C$, by the rules in Fig. 4. Note that $\Delta$ is also synthesized as well as $\tau$ and $C$ in this step. This difference in the treatment of $\Gamma$ and $\Delta$ is why we separate multiplicity environments $\Delta$ from typing environments $\Gamma$.

Gathered constraints are solved when we process top-level bindings. Figure 5 defines *type inference judgment for programs*, $\Gamma \blacktriangleright prog$, which reads that the inference finds *prog* well-typed under $\Gamma$. In the rules, manipulation of constraints is done by the *simplification judgment* $Q \blacktriangleright_{\mathrm{simp}} C \rightsquigarrow Q'; \theta$, which simplifies $C$ under the assumption $Q$ into the pair $(Q', \theta)$ of residual constraints $Q'$ and substitution $\theta$ for unification variables, where $(Q', \theta)$ is expected to be equivalent

$$\frac{\Gamma(x) = \forall \overline{p a}.Q \Rightarrow \tau \quad \overline{\alpha}, \overline{\pi} : \text{fresh}}{\Gamma \Vdash x : \tau[\overline{p \mapsto \pi}, \overline{a \mapsto \alpha}] \rightsquigarrow x^1; Q[\overline{p \mapsto \pi}]} \qquad \frac{\Gamma, x : \alpha \Vdash e : \tau \rightsquigarrow \Delta, x^M; C \quad \alpha, \pi : \text{fresh}}{\Gamma \Vdash \lambda x.e : \alpha \to_\pi \tau \rightsquigarrow \Delta; C \wedge M \leq \pi}$$

$$\frac{\Gamma \Vdash e_1 : \tau_1 \rightsquigarrow \Delta_1; C_2 \quad \Gamma \Vdash e_2 : \tau_2 \rightsquigarrow \Delta_2; C_1 \quad \beta, \pi : \text{fresh}}{\Gamma \Vdash e_1\ e_2 : \beta \rightsquigarrow \Delta_1 + \pi\Delta_2; C_1 \wedge C_2 \wedge \tau_1 \sim (\tau_2 \to_\pi \beta)}$$

$$\frac{\mathsf{C} : \forall \overline{p a}.\ \overline{\sigma} \to_{\overline{\nu}} \mathsf{D}\ \overline{p}\ \overline{a} \quad \{\Gamma \Vdash e_i : \tau_i \rightsquigarrow \Delta_i; C_i\}_i \quad \overline{\alpha}, \overline{\pi} : \text{fresh}}{\Gamma \Vdash \mathsf{C}\ \overline{e} : \mathsf{D}\ \overline{\pi}\ \overline{\alpha} \rightsquigarrow \sum_i \nu_i[\overline{p \mapsto \pi}]\Delta_i; \bigwedge_i C_i \wedge \tau_i \sim \sigma_i[\overline{p \mapsto \pi}, \overline{a \mapsto \alpha}]}$$

$$\frac{\begin{array}{l} \Gamma \Vdash e_0 : \tau_0 \rightsquigarrow \Delta_0; C_0 \quad \pi_0, \overline{\pi_i}, \overline{\alpha_i}, \beta : \text{fresh} \\ \left\{ \dfrac{\mathsf{C}_i : \forall \overline{p a}.\ \overline{\tau_i} \to_{\overline{\nu_i}} \mathsf{D}\ \overline{p}\ \overline{a}}{\Gamma, \overline{x_i : \tau_i[p \mapsto \pi_i, a \mapsto \alpha_i]} \Vdash e_i : \tau_i' \rightsquigarrow \Delta_i, \overline{x_i^{M_i}}; C_i} \right\}_i \\ C' = C_0 \wedge \bigwedge_i \left( C_i \wedge \beta \sim \tau_i' \wedge (\tau_0 \sim \mathsf{D}\ \overline{\pi_i}\ \overline{\alpha_i}) \wedge \bigwedge_j M_{ij} \leq \pi_0 \nu_{ij}[p \mapsto \pi_i] \right) \end{array}}{\Gamma \Vdash \textbf{case}\ e_0\ \textbf{of}\ \{\mathsf{C}_i\ \overline{x_i} \to e_i\}_i : \beta \rightsquigarrow \pi_0\Delta_0 + \bigsqcup_i \Delta_i; C'}$$

**Fig. 4.** Type inference rules for expressions

$$\frac{}{\Gamma \Vdash \varepsilon} \qquad \frac{\begin{array}{c}\Gamma \Vdash e : \tau \rightsquigarrow \Delta; C \quad \top \Vdash_{\text{simp}} C \rightsquigarrow Q; \theta \quad \{\overline{\pi\alpha}\} = \mathsf{fuv}(Q, \tau\theta) \\ \overline{p}, \overline{a} : \text{fresh} \quad \Gamma, f : \forall \overline{pa}.(Q \Rightarrow \tau\theta)[\overline{\alpha \mapsto a, \pi \mapsto p}] \Vdash \mathit{prog}\end{array}}{\Gamma \Vdash f = e; \mathit{prog}}$$

$$\frac{\Gamma \Vdash e : \sigma \rightsquigarrow \Delta; C \quad Q \Vdash_{\text{simp}} C \wedge \tau \sim \sigma \rightsquigarrow \top; \theta \quad \Gamma, f : \forall \overline{pa}.Q \Rightarrow \tau \Vdash \mathit{prog}}{\Gamma \Vdash f : (\forall \overline{pa}.Q \Rightarrow \tau) = e; \mathit{prog}}$$

**Fig. 5.** Type inference rules for programs in some sense to $C$ under the assumption $Q$. The idea underlying our simplification is to solve type equality constraints in $C$ as much as possible and then remove predicates that are implied by $Q$. Rules S-Fun, S-Data, S-Uni, and S-Triv are responsible for the former, which decompose type equality constraints and yield substitutions once either of the sides becomes a unification variable. Rules S-Entail and S-Rem are responsible for the latter, which remove predicates implied by $Q$ and then return the residual constraints. Rule S-Entail checks $Q \models \phi$; a concrete method for this check will be discussed in Sect. 3.2.

*Example 1 (app).* Let us illustrate how the system infers a type for $app = \lambda f.\lambda x.f\ x$. We have the following derivation for its body $\lambda f.\lambda x.f\ x$:

$$\dfrac{\dfrac{\dfrac{\dfrac{}{f : \alpha_f \Vdash f : \alpha_f \rightsquigarrow f^1; \top} \quad \dfrac{}{x : \alpha_x \Vdash x : \alpha_x \rightsquigarrow x^1; \top}}{f : \alpha_f, x : \alpha_x \Vdash f\ x : \beta \rightsquigarrow f^1, x^\pi; \alpha_f \sim (\alpha_x \to_\pi \beta)}}{f : \alpha_f \Vdash \lambda x.f\ x : \alpha_x \to_{\pi_x} \beta \rightsquigarrow f^1; \alpha_f \sim (\alpha_x \to_\pi \beta) \wedge \pi_x \leq \pi}}{\Vdash \lambda f.\lambda x.f\ x : \alpha_f \to_{\pi_f} \alpha_x \to_{\pi_x} \beta \rightsquigarrow \emptyset; \alpha_f \sim (\alpha_x \to_\pi \beta) \wedge \pi_x \leq \pi \wedge 1 \leq \pi_f}$$

The highlights in the above derivation are:

– In the last two steps, $f$ is assigned to type $\alpha_f$ and multiplicity $\pi_f$, and $x$ is assigned to type $\alpha_x$ and multiplicity $\pi_x$.

$$\frac{Q \Vdash_{\rm simp} \sigma \sim \sigma' \wedge \mu \le \mu' \wedge \mu' \le \mu \wedge \tau \sim \tau' \rightsquigarrow Q'; \theta}{Q \Vdash_{\rm simp} (\sigma \to_\mu \tau) \sim (\sigma' \to_{\mu'} \tau') \wedge C \rightsquigarrow Q'; \theta}\ \textsc{S-Fun}$$

$$\frac{Q \Vdash_{\rm simp} \overline{\mu \le \mu'} \wedge \overline{\mu' \le \mu} \wedge \overline{\sigma \sim \sigma'} \wedge C \rightsquigarrow Q'; \theta}{Q \Vdash_{\rm simp} (\mathsf{D}\ \overline{\mu}\ \overline{\sigma}) \sim (\mathsf{D}\ \overline{\mu'}\ \overline{\sigma'}) \wedge C \rightsquigarrow Q'; \theta}\ \textsc{S-Data}$$

$$\frac{\alpha \notin \mathsf{fuv}(\tau) \quad Q \Vdash_{\rm simp} C[\alpha \mapsto \tau] \rightsquigarrow Q'; \theta}{Q \Vdash_{\rm simp} \alpha \sim \tau \wedge C \rightsquigarrow Q'; \theta \circ [\alpha \mapsto \tau]}\ \textsc{S-Uni} \qquad \frac{Q \Vdash_{\rm simp} C \rightsquigarrow Q'; \theta}{Q \Vdash_{\rm simp} \tau \sim \tau \wedge C \rightsquigarrow Q'; \theta}\ \textsc{S-Triv}$$

$$\frac{Q \wedge Q_{\rm w} \models \phi \quad Q \Vdash_{\rm simp} Q_{\rm w} \wedge C \rightsquigarrow Q'; \theta}{Q \Vdash_{\rm simp} \phi \wedge Q_{\rm w} \wedge C \rightsquigarrow Q'; \theta}\ \textsc{S-Entail} \qquad \frac{\text{no other rules can apply}}{Q \Vdash_{\rm simp} Q' \rightsquigarrow Q'; \emptyset}\ \textsc{S-Rem}$$

**Fig. 6.** Simplification rules (modulo commutativity and associativity of $\wedge$ and commutativity of $\sim$)

- Then, in the third last step, for $f\ x$, the system infers type $\beta$ with constraint $\alpha_f \sim (\alpha_x \to_\pi \beta)$. At the same time, the variable use in $f\ x$ is also inferred as $f^1, x^\pi$. Note that the use of $x$ is $\pi$ because it is passed to $f : \alpha_x \to_\pi \beta$.
- After that, in the last two steps again, the system yields constraints $\pi_x \le \pi$ and $1 \le \pi_f$.

As a result, the type $\tau = \alpha_f \to_{\pi_f} \alpha_x \to_{\pi_x} \beta$ is inferred with the constraint $C = \alpha_f \sim (\alpha_x \to_\pi \beta) \wedge \pi_x \le \pi \wedge 1 \le \pi_f$.

Then, we try to assign a polytype to *app* by the rules in Fig. 4. By simplification, we have $\top \Vdash_{\rm simp} C \rightsquigarrow \pi_x \le \pi; [\alpha_f \mapsto (\alpha_x \to_\pi \beta)]$. Thus, by generalizing $\tau[\alpha_f \mapsto (\alpha_x \to_\pi \beta)] = (\alpha_x \to_\pi \beta) \to_{\pi_f} \alpha_x \to_{\pi_x} \beta$ with $\pi_x \le \pi$, we obtain the following type for *app*:

$$app : \forall p\, p_f\, p_x\, a\, b.\ p \le p_x \Rightarrow (a \to_p b) \to_{p_f} a \to_{p_x} b \qquad \square$$

*Correctness* We first prepare some definitions for the correctness discussions. First, we allow substitutions $\theta$ to replace unification multiplicity variables as well as unification type variables. Then, we extend the notion of $\models$ and write $C \models C'$ if $C'\theta$ holds when $C\theta$ holds. From now on, we require that substitutions are idempotent, i.e., $\tau\theta\theta = \tau\theta$ for any $\tau$, which excludes substitutions $[\alpha \mapsto \mathsf{List}\ \alpha]$ and $[\alpha \mapsto \beta, \beta \mapsto \mathsf{Int}]$ for example. Let us write $Q \models \theta = \theta'$ if $Q \models \tau\theta \sim \tau\theta'$ for any $\tau$. The restriction of a substitution $\theta$ to a domain $X$ is written by $\theta|_X$.

Consider a pair $(Q_{\rm g}, C_{\rm w})$, where we call $Q_{\rm g}$ and $C_{\rm w}$ given and wanted constraints, respectively. Then, a pair $(Q, \theta)$ is called a (sound) *solution* [31] for the pair $(Q_{\rm g}, C_{\rm w})$ if $Q_{\rm g} \wedge Q \models C_{\rm w}\theta$, $\mathsf{dom}(\theta) \cap \mathsf{fuv}(Q_{\rm g}) = \emptyset$, and $\mathsf{dom}(\theta) \cap \mathsf{fuv}(Q) = \emptyset$. A solution is called *guess-free* [31] if it satisfies $Q_{\rm g} \wedge C_{\rm w} \models Q \wedge \bigwedge_{\pi \in \mathsf{dom}(\theta)} (\pi = \theta(\pi)) \wedge \bigwedge_{\alpha \in \mathsf{dom}(\theta)} (\alpha \sim \theta(\alpha))$ in addition. Intuitively, a guess-free solution consists of necessary conditions required for a wanted constraint $C_{\rm w}$ to hold, assuming a given constraint $Q_{\rm g}$. For example, for $(\top, \alpha \sim (\beta \to_1 \beta))$, $(\top, [\alpha \mapsto (\mathsf{Int} \to_1 \mathsf{Int}), \beta \mapsto \mathsf{Int}])$ is a solution but not guess-free. Very roughly speaking, being for $(Q, \theta)$ a guess-free solution of $(Q_{\rm g}, C_{\rm w})$ means that $(Q, \theta)$ is equivalent to $C_{\rm w}$ under the assumption $Q_{\rm g}$. There can be multiple guess-free solutions; for example, for $(\top, \pi \le 1)$, both $(\pi \le 1, \emptyset)$ and $(\top, [\pi \mapsto 1])$ are guess-free solutions.

**Lemma 10 (Soundness and Principality of Simplification).** If $Q \mapsto_{\mathrm{simp}} C \rightsquigarrow Q'; \theta$, $(Q', \theta)$ is a guess-free solution for $(Q, C)$. □

**Lemma 11 (Completeness of Simplification).** If $(Q', \theta)$ is a solution for $(Q, C)$ where $Q$ is satisfiable, then $Q \mapsto_{\mathrm{simp}} C \rightsquigarrow Q''; \theta'$ for some $Q''$ and $\theta'$. □

**Theorem 2 (Soundness of Inference).** Suppose $\Gamma \mapsto e : \tau \rightsquigarrow \Delta; C$ and there is a solution $(Q, \theta)$ for $(\top, C)$. Then, we have $Q; \Gamma\theta; \Delta\theta \vdash e : \tau\theta$. □

**Theorem 3 (Completeness and Principality of Inference).** Suppose $\Gamma \mapsto e : \tau \rightsquigarrow \Delta; C$. Suppose also that $Q'; \Gamma\theta'; \Delta' \vdash e : \tau'$ for some substitution $\theta'$ on unification variables such that $\mathsf{dom}(\theta') \subseteq \mathsf{fuv}(\Gamma)$ and $\mathsf{dom}(\theta') \cap \mathsf{fuv}(Q') = \emptyset$. Then, there exists $\theta$ such that $\mathsf{dom}(\theta) \setminus \mathsf{dom}(\theta') \subseteq X$, $(Q', \theta)$ is a solution for $(\top, C)$, $Q' \models \theta|_{\mathsf{dom}(\theta')} = \theta'$, $Q' \models \tau\theta \sim \tau'$, and $Q' \models \Delta\theta \leq \Delta'$, where $X$ is the set of unification variables introduced in the derivation. □

Note that the constraint generation $\Gamma \mapsto e : \tau \rightsquigarrow \Delta; C$ always succeeds, whereas the generated constraints may possibly be conflicting. Theorem 3 states that such a case cannot happen when $e$ is well-typed under the rules in Fig. 2.

*Incompleteness in Typing Programs.* It may sound contradictory to Theorem 3, but the type inference is indeed incomplete for checking type-annotated bindings. Recall that the typing rule for type-annotated bindings requires that the resulting constraint after simplification must be $\top$. However, even when there exists a solution of the form $(\top, \theta)$ for $(Q, C)$, there can be no guess-free solution of this form. For example, $(\top, \pi \leq \pi')$ has a solution $(\top, [\pi \mapsto \pi'])$, but there are no guess-free solutions of the required form. Also, even though there exists a guess-free solution of the form $(\top, \theta)$, the simplification may not return the solution, as guess-free solutions are not always unique. For example, for $(\top, \pi \leq \pi' \wedge \pi' \leq \pi)$, $(\top, [\pi \mapsto \pi'])$ is a guess-free solution, whereas we have $\top \mapsto_{\mathrm{simp}} \pi \leq \pi' \wedge \pi' \leq \pi \rightsquigarrow \pi \leq \pi' \wedge \pi' \leq \pi; \emptyset$. The source of the issue is that constraints on multiplicities can (also) be solved by substitutions.

Fortunately, this issue disappears when we consider disambiguation in Sect. 4. By disambiguation, we can eliminate constraints for internally-introduced multiplicity unification variables that are invisible from the outside. As a result, after processing equality constraints, we essentially need only consider rigid multiplicity variables when checking entailment for annotated top-level bindings.

*Promoting Equalities to Substituions.* The inference can infer polytypes $\forall p.\ p \leq 1 \Rightarrow \mathsf{Int} \to_p \mathsf{Int}$ and $\forall p_1 p_2.\ (p_1 \leq p_2 \wedge p_2 \leq p_1) \Rightarrow \mathsf{Int} \to_{p_1} \mathsf{Int} \to_{p_2} \mathsf{Int}$, while programmers would prefer more simpler types $\mathsf{Int} \to_1 \mathsf{Int}$ and $\forall p.\ \mathsf{Int} \to_p \mathsf{Int} \to_p \mathsf{Int}$; the simplification so far does not yield substitutions on multiplicity unification variables. Adding the following rule remedies the situation:

$$\frac{\pi \notin \mathsf{fuv}(Q) \quad \pi \neq \mu \quad Q \wedge Q_{\mathrm{w}} \models \pi \leq \mu \wedge \mu \leq \pi \quad Q \mapsto_{\mathrm{simp}} (Q_{\mathrm{w}} \wedge C)[\pi \mapsto \mu] \rightsquigarrow Q'; \theta}{Q \mapsto_{\mathrm{simp}} Q_{\mathrm{w}} \wedge C \rightsquigarrow Q'; \theta \circ [\pi \mapsto \mu]}\ \textsc{S-Eq}$$

This rule says that if $\pi = \mu$ must hold for $Q_{\mathrm{w}} \wedge C$ to hold, the simplification yields the substitution $[\pi \mapsto \mu]$. The condition $\pi \notin \mathsf{fuv}(Q)$ is required for Lemma 10; a solution cannot substitute variables in $Q$. Note that this rule essentially finds an improving substitution [16].

Using the rule is optional. Our prototype implementation actually uses S-Eq only for $Q_{\mathrm{w}}$ for which we can find $\mu$ easily: $M \leq 1$, $\omega \leq \mu$, and looping chains $\mu_1 \leq \mu_2 \wedge \cdots \wedge \mu_{n-1} \leq \mu_n \wedge \mu_n \leq \mu_1$.

#### 3.2 Entailment Checking by Horn SAT Solving

The simplification rules rely on the check of entailment $Q \models \phi$. For the constraints in this system, we can perform this check in quadratic time at worst but in linear time for most cases. Specifically, we reduce the checking $Q \models \phi$ to satisfiability of propositional Horn formulas (Horn SAT), which is known to be solved in linear time in the number of occurrences of literals [10], where the reduction (precisely, the preprocessing of the reduction) may increase the problem size quadratically. The idea of using Horn SAT for constraint solving in linear typing can be found in Mogensen [23].

First, as a preprocess, we normalize both given and wanted constraints by the following rules:

- Replace $M_1 \cdot M_2 \leq M$ with $M_1 \leq M \wedge M_2 \leq M$.
- Replace $M \cdot 1$ and $1 \cdot M$ with $M$, and $M \cdot \omega$ and $\omega \cdot M$ with $\omega$.
- Remove trivial predicates $1 \leq M$ and $M \leq \omega$.

After this, each predicate $\phi$ has the form $\mu \leq \prod_i \nu_i$.

After the normalization above, we can reduce the entailment checking to satisfiability. Specifically, we use the following property:

$$Q \models \mu \leq \prod_i \nu_i \quad \text{iff} \quad Q \wedge \bigwedge_i (\nu_i \leq 1) \wedge (\omega \leq \mu) \text{ is unsatisfiable}$$

Here, the constraint $Q \wedge \bigwedge_i (\nu_i \leq 1) \wedge (\omega \leq \mu)$ intuitively asserts that there exists a counterexample of $Q \models \mu \leq \prod_i \nu_i$.

Then, it is straightforward to reduce the satisfiability of $Q$ to Horn SAT; we just map 1 to true and $\omega$ to false and accordingly map $\leq$ and $\cdot$ to $\Leftarrow$ and $\wedge$, respectively. Since Horn SAT can be solved in linear time in the number of occurrences of literals [10], the reduction also shows that the satisfiability of $Q$ is checked in linear time in the size of $Q$ if $Q$ is normalized.

**Corollary 1.** Checking $Q \models \phi$ is in linear time if $Q$ and $\phi$ are normalized. □

The normalization of constraints can duplicate $M$ of $\cdots \leq M$, and thus increases the size quadratically in the worst case. Fortunately, the quadratic increase is not common because the size of $M$ is bounded in practice, in many cases by one. Among the rules in Fig. 2, only the rule that introduces non-singleton $M$ in the right-hand side of $\leq$ is Case for a constructor whose arguments'

multiplicities are non-constants, such as $\mathsf{MkMany} : \forall p\, a.\, a \to_p \mathsf{Many}\ p\ a$. However, it often suffices to use non-multiplicity-parameterized constructors, such as $\mathsf{Cons} : \forall a.\ a \to_1 \mathsf{List}\ a \to_1 \mathsf{List}\ a$, because such constructors can be used to construct or deconstruct both linear and unrestricted data.

#### 3.3 Issue: Inference of Ambiguous Types

The inference system so far looks nice; the system is sound and complete, and infers principal types. However, there still exists an issue to overcome for the system to be useful: it often infers ambiguous types [15, 27] in which internal multiplicity variables leak out to reveal internal implementation details.

Consider $app' = \lambda f.\lambda x.app\ f\ x$ for $app = \lambda f.\lambda x.f\ x$ from Example 1. We would expect that equivalent types are inferred for $app'$ and $app$. However, this is not the case for the inference system. In fact, the system infers the following type for $app'$ (here we reproduce the inferred type of $app$ for comparison):

$$\begin{array}{ll} app\ : & \forall p\, p_f\, p_x\, a\, b.\ (p \le p_x) \Rightarrow (a \to_p b) \to_{p_f} a \to_{p_x} b \\ app' : & \forall q\, q_f\, q_x\, p_f\, p_x\, a\, b.\ (q \le q_x \wedge q_f \le p_f \wedge q_x \le p_x) \Rightarrow (a \to_q b) \to_{p_f} a \to_{p_x} b \end{array}$$

We highlight why this type is inferred as follows.

- By abstractions, $f$ is assigned to type $\alpha_f$ and multiplicity $\pi_f$, and $x$ is assigned to type $\alpha_x$ and multiplicity $\pi_x$.
- By its use, $app$ is instantiated to type $(\alpha' \to_{\pi'} \beta') \to_{\pi'_f} \alpha' \to_{\pi'_x} \beta'$ with constraint $\pi' \le \pi'_x$.
- For $app\ f$, the system infers type $\beta$ with constraint $((\alpha' \to_{\pi'} \beta') \to_{\pi'_f} \alpha' \to_{\pi'_x} \beta') \sim (\alpha_f \to_{\pi_1} \beta)$. At the same time, the variable use in the expression is inferred as $app^1, f^{\pi_1}$.
- For $(app\ f\ x)$, the system infers type $\gamma$ with constraint $\beta \sim (\alpha' \to_{\pi_2} \gamma)$. At the same time, the variable use in the expression is inferred as $app^1, f^{\pi_1}, x^{\pi_2}$.
- As a result, $\lambda f.\lambda x.app\ f\ x$ has type $\alpha_f \to_{\pi_f} \alpha_x \to_{\pi_x} \gamma$, yielding constraints $\pi_1 \le \pi_f \wedge \pi_2 \le \pi_x$.

Then, for the gathered constraints, by simplification (including S-Eq), we obtain a (guess-free) solution $(Q, \theta)$ such that $Q = (\pi'_f \le \pi_f \wedge \pi' \le \pi'_x \wedge \pi'_x \le \pi_x)$ and $\theta = [\alpha_f \mapsto (\alpha' \to_{\pi'} \beta'), \pi'_1 \mapsto \pi'_f, \beta \mapsto (\alpha_f \to_{\pi'_x} \beta'), \pi_2 \mapsto \pi'_x, \gamma \mapsto \beta'])$. Then, after generalizing $(\alpha_f \to_{\pi_f} \alpha_x \to_{\pi_x} \gamma)\theta = (\alpha' \to_{\pi'} \beta') \to_{\pi_f} \alpha' \to_{\pi_x} \beta$, we obtain the inferred type above.

There are two problems with this inference result:

- The type of $app'$ is *ambiguous* in the sense that the type-level variables in the constraint cannot be determined only by those that appear in the type [15,27]. Usually, ambiguous types are undesirable, especially when their instantiation affects runtime behavior [15, 27, 31].
- Due to this ambiguity, the types of $app$ and $app'$ are not judged equivalent by the inference system. For example, the inference rejects the binding $app'' : \forall p\, p_f\, p_x\, a\, b.\ (p \le p_x) \Rightarrow (a \to_p b) \to_{p_f} a \to_{p_x} b = app'$ because the system does not know how to instantiate the ambiguous type-level variables $q_f$ and $q_x$, while the binding is valid in the type system in Sect. 2.

Inference of ambiguous types is common in the system; it is easily caused by using defined variables. Rejecting ambiguous types is not a solution for our case because it rejects many programs. Defaulting such ambiguous type-level variables to 1 or $\omega$ is not a solution either because it loses principality in general. However, we have no other choices than to reject ambiguous types, *as long as multiplicities are relevant in runtime behavior.*

In the next section, we will show how we address the ambiguity issue under the assumption that multiplicities are irrelevant at runtime. Under this assumption, it is no problem to have multiplicity-monomorphic primitives such as array processing primitives (e.g., $\mathit{readMArray} : \forall a.\, \mathsf{MArray}\ a \to_1 \mathsf{Int} \to_\omega (\mathsf{MArray}\ a \otimes \mathsf{Un}\ a)$) [31]. Note that this assumption does not rule out all multiplicity-polymorphic primitives; it just prohibits the primitives from inspecting multiplicities at runtime.

## 4 Disambiguation by Quantifier Elimination

In this section, we address the issue of ambiguous and leaky types by using quantifier elimination. The basic idea is simple; we just view the type of $\mathit{app}'$ as

$$\mathit{app}' : \forall q\, p_f\, p_x\, a\, b.\ (\exists q_x\, q_f.\ q \le q_x \wedge q_f \le p_f \wedge q_x \le p_x) \Rightarrow (a \to_q b) \to_{p_f} a \to_{p_x} b$$

In this case, the constraint $(\exists q_x\, q_f.\ q \le q_x \wedge q_f \le p_f \wedge q_x \le p_x)$ is logically equivalent to $q \le p_x$, and thus we can infer the equivalent types for both $\mathit{app}$ and $\mathit{app}'$. Fortunately, such quantifier elimination is always possible for our representation of constraints; that is, for $\exists p.Q$, there always exists $Q'$ that is logically equivalent to $\exists p.Q$. A technical subtlety is that, although we perform quantifier elimination after generalization in the above explanation, we actually perform quantifier elimination just before generalization, or more precisely, as a final step of simplification, for compatibility with the simplification in OutsideIn(X) [31], especially in the treatment of local assumptions.

### 4.1 Elimination of Existential Quantifiers

The elimination of existential quantifiers is rather easy; we simply use the well-known fact that a disjunction of a Horn clause and a definite clause can also be represented as a Horn clause. Regarding our encoding of normalized predicates (Sect. 3.2) that maps $\mu \le M$ to a Horn clause, the fact can be rephrased as:

**Lemma 12.** $(\mu \le M \vee \omega \le M') \equiv \mu \le M \cdot M'$. □

Here, we extend constraints to include $\vee$ and write $\equiv$ for the logical equivalence; that is, $Q \equiv Q'$ if and only if $Q \models Q'$ and $Q' \models Q$.

As a corollary, we obtain the following result:

**Corollary 2.** There effectively exists a quantifier-free constraint $Q'$, denoted by $\mathsf{elim}(\exists \pi.Q)$, such that $Q'$ is logically equivalent to $\exists \pi.Q$.

*Proof.* Note that $\exists\pi.Q$ means $Q[\pi \mapsto 1] \vee Q[\pi \mapsto \omega]$ because $\pi$ ranges over $\{1, \omega\}$. We safely assume that $Q$ is normalized (Sect. 3.2) and that $Q$ does not contain a predicate $\pi \leq M$ where $\pi$ appears also in $M$, because such a predicate trivially holds.

We define $\Phi_1$, $\Phi_\omega$, and $Q_{\mathrm{rest}}$ as $\Phi_1 = \{\mu \leq M \mid (\mu \leq \pi \cdot M) \in Q, \mu \neq \pi\}$, $\Phi_\omega = \{\omega \leq M \mid (\pi \leq M) \in Q, \pi \notin \mathsf{fuv}(M)\}$, and $Q_{\mathrm{rest}} = \bigwedge \{\phi \mid \phi \in Q, \pi \notin \mathsf{fuv}(\phi)\}$. Here, we abused the notation to write $\phi \in Q$ to mean that $Q = \bigwedge_i \phi_i$ and $\phi = \phi_i$ for some $i$. In the construction of $\Phi_1$, we assumed the monoid laws of $(\cdot)$; the definition says that we remove $\pi$ from the right-hand sides and $M$ becomes 1 if the right-hand side is $\pi$. By construction, $Q[p \mapsto 1]$ and $Q[p \mapsto \omega]$ are equivalent to $(\bigwedge \Phi_1) \wedge Q_{\mathrm{rest}}$ and $(\bigwedge \Phi_\omega) \wedge Q_{\mathrm{rest}}$, respectively. Thus, by Lemma 12 and by the distributivity of $\vee$ over $\wedge$ it suffices to define $Q'$ as $Q' = (\bigwedge \{\mu \leq M \cdot M' \mid \mu \leq M \in \Phi_1, \omega \leq M' \in \Phi_\omega\}) \wedge Q_{\mathrm{rest}}$. □

*Example 2.* Consider $Q = (\pi'_f \leq \pi_f \wedge \pi' \leq \pi'_x \wedge \pi'_x \leq \pi_x)$; this is the constraint obtained from $\lambda f.\lambda x.app\ f\ x$ (Sect. 3.3). Since $\pi'_f$ and $\pi'_x$ do not appear in the inferred type $(\alpha' \to_{\pi'} \beta') \to_{\pi_f} \alpha' \to_{\pi_x} \beta$, we want to eliminate them by the above step. There is a freedom to choose which variable is eliminated first. Here, we shall choose $\pi'_f$ first.

First, we have $\mathsf{elim}(\exists\pi'_f.Q) = \pi' \leq \pi'_x \wedge \pi'_x \leq \pi_x$ because for this case we have $\Phi_1 = \emptyset$, $\Phi_\omega = \{\omega \leq \pi_f\}$, and $Q_{\mathrm{rest}} = \pi' \leq \pi'_x \wedge \pi'_x \leq \pi_x$. We then have $\mathsf{elim}(\exists\pi'_x.\pi' \leq \pi'_x \wedge \pi'_x \leq \pi_x) = \pi' \leq \pi_x$ because for this case we have $\Phi_1 = \{\pi' \leq 1\}$, $\Phi_2 = \{\omega \leq \pi_x\}$, and $Q_{\mathrm{rest}} = \top$. □

In the worst case, the size of $\mathsf{elim}(\exists\pi.Q)$ can be quadratic to that of $Q$. Thus, repeating elimination can make the constraints exponentially bigger. We believe that such blow-up rarely happens because it is usual that $\pi$ occurs only in a few predicates in $Q$. Also, recall that non-singleton right-hand sides are caused only by multiplicity-parameterized constructors. When each right-hand side of $\leq$ is a singleton in $Q$, the same holds in $\mathsf{elim}(\exists\pi.Q)$. For such a case, the exponential blow-up cannot happen because the size of constraints in the form is at most quadratic in the number of multiplicity variables.

#### 4.2 Modified Typing Rules

As mentioned at the begging of this section, we perform quantifier elimination as the last step of simplification. To do so, we define $Q \mapsto^{\tau}_{\mathrm{simp}} C \rightsquigarrow Q''; \theta$ as follows:

$$\frac{Q \mapsto_{\mathrm{simp}} C \rightsquigarrow Q'; \theta \quad \{\overline{\pi}\} = \mathsf{fuv}(Q') \setminus \mathsf{fuv}(\tau\theta) \quad Q'' = \mathsf{elim}(\exists\overline{\pi}.Q')}{Q \mapsto^{\tau}_{\mathrm{simp}} C \rightsquigarrow Q''; \theta}$$

Here, $\tau$ is used to determine which unification variables will be ambiguous after generalization. We simply identify variables ($\overline{\pi}$ above) that are not in $\tau$ as ambiguous [15] for simplicity. This check is indeed conservative in a more general definition of ambiguity [27], in which $\forall p\, r\, a. (p \leq r, r \leq p) \Rightarrow a \to_p a$ for example is not judged as ambiguous because $r$ is determined by $p$.

Then, we replace the original simplification with the above-defined version.

$$\frac{\Gamma \Vdash e : \tau \rightsquigarrow \Delta; C \quad \boxed{\top \Vdash^{\tau}_{\mathrm{simp}} C \rightsquigarrow Q; \theta} \quad \{\overline{\pi\alpha}\} = \mathsf{fuv}(Q, \tau\theta) \quad \overline{p}, \overline{a} : \text{fresh} \quad \Gamma, f : \forall \overline{pa}.(Q \Rightarrow \tau\theta)[\overline{\alpha \mapsto a, \pi \mapsto p}] \Vdash \mathit{prog}}{\Gamma \Vdash f = e; \mathit{prog}}$$

$$\frac{\Gamma \Vdash e : \sigma \rightsquigarrow \Delta; C \quad \boxed{Q \Vdash^{\sigma}_{\mathrm{simp}} C \wedge \tau \sim \sigma \rightsquigarrow \top; \theta} \quad \Gamma, f : \forall \overline{pa}.Q \Rightarrow \tau \Vdash \mathit{prog}}{\Gamma \Vdash f : (\forall \overline{pa}.Q \Rightarrow \tau) = e; \mathit{prog}}$$

Here, the changed parts are highlighted for readability.

*Example 3.* Consider $(Q, \theta)$ in Sect. 3.3 such that $Q = (\pi'_f \leq \pi_f \wedge \pi' \leq \pi'_x \wedge \pi'_x \leq \pi_x)$ and $\theta = [\alpha_f \mapsto (\alpha' \to_{\pi'} \beta'), \pi'_1 \mapsto \pi'_f, \beta \mapsto (\alpha_f \to_{\pi'_x} \beta'), \pi_2 \mapsto \pi'_x, \gamma \mapsto \beta']$), which is obtained after simplification of the gathered constraint. Following Example 2, eliminating variables that are not in $\tau\theta = (\alpha' \to_{\pi'} \beta') \to_{\pi_f} \alpha' \to_{\pi_x} \beta$ yields the constraint $\pi' \leq \pi_x$. As a result, by generalization, we obtain the polytype

$$\forall q\, p_f\, p_x\, a\, b.\ (q \leq p_x) \Rightarrow (a \to_q b) \to_{p_f} a \to_{p_x} b$$

for $\mathit{app}'$, which is equivalent to the inferred type of $\mathit{app}$. □

Note that $(Q', \theta)$ of $Q \Vdash^{\tau}_{\mathrm{simp}} C \rightsquigarrow Q'; \theta$ is no longer a solution of $(Q, C)$ because $C$ can have eliminated variables. However, it is safe to use this version when generalization takes place, because, for variables $\overline{q}$ that do not occur in $\tau$, $\forall \overline{pqa}.\ Q \Rightarrow \tau$ and $\forall \overline{pa}.\ Q' \Rightarrow \tau$ have the same set of monomorphic instances, if $\exists \overline{q}.Q$ is logically equivalent to $Q'$. Note that in this type system simplification happens only before (implicit) generalization takes place.

### 5 Extension to Local Assumptions

In this section, following OutsideIn(X) [31], we extend our system with local assumptions, which enable us to have **let**s and GADTs. We focus on the treatment of **let**s in this section because type inference for **let**s involves a linearity-specific concern: the multiplicity of a **let**-bound variable.

#### 5.1 "Let Should Not Be Generalized" for Our Case

We first discuss that even for our case "**let** should not be generalized" [31]. That is, generalization of **let** sometimes results in counter-intuitive typing and conflicts with the discussions so far.

Consider the following program:

$$h = \lambda f.\lambda k.\mathbf{let}\ y = f\ (\lambda x.k\ x)\ \mathbf{in}\ 0$$

Suppose for simplicity that $f$ and $x$ have types $(a \to_{\pi_1} b) \to_{\pi_2} c$ and $a \to_{\pi_3} b$, respectively (here we only focus on the treatment of multiplicity). Then, $f\ (\lambda x.k\ x)$

has type $c$ with the constraint $\pi_3 \le \pi_1$. Thus, after generalization, $y$ has type $\pi_3 \le \pi_1 \Rightarrow c$, where $\pi_3$ and $\pi_1$ are neither generalized nor eliminated because they escape from the definition of $y$. As a result, $h$ has type $\forall p_1\, p_2\, p_3\, a\, b\, c. ((a \to_{p_1} b) \to_{p_2} c) \to_\omega (a \to_{p_3} b) \to_\omega \mathsf{Int}$; there is no constraint $p_3 \le p_1$ because the definition of $y$ does not yield a constraint. This nonexistence of the constraint would be counter-intuitive because users wrote $f\ (\lambda x.k\ x)$ while the constraint for the expression is not imposed. In particular, it does not cause an error even when $f : (a \to_1 b) \to_1 c$ and $k : a \to_\omega b$, while $f\ (\lambda x.k\ x)$ becomes illegal for this case. Also, if we change 0 to $y$, the error happens at the use site instead of the definition site. Moreover, the type is fragile as it depends on whether $y$ occurs or not; for example, if we change 0 to $const\ 0\ y$ where $const = \lambda a.\lambda b.a$, the type of $h$ changes to $\forall p_1\, p_2\, p_3\, a\, b\, c.\, p_1 \le p_3 \Rightarrow ((a \to_{p_1} b) \to_{p_2} c) \to_\omega (a \to_{p_3} b) \to_\omega \mathsf{Int}$. In this discussion, we do not consider type-equality constraints, but there are no legitimate reasons why type-equality constraints are solved on the fly in typing $y$.

As demonstrated in the above example, "**let** should not be generalized" [30,31] in our case. Thus, we adopt the same principle in OutsideIn(X) that **let** will be generalized only if users write a type annotation for it [31]. This principle is also adopted in GHC (as of 6.12.1 when the language option `MonoLocalBinds` is turned on) with a slight relaxation to generalize closed bindings.

#### 5.2 Multiplicity of Let-Bound Variables

Another issue with **let**-generalization, which is specific to linear typing, is that a generalization result depends on the multiplicity of the **let**-bound variable. Let us consider the following program, where we want to generalize the type of $y$ (even without a type annotation):

$$g = \lambda x.\mathbf{let}\ y = \lambda f.f\ x\ \mathbf{in}\ y\ not$$

Suppose for simplicity that $\mathsf{not}$ has type $\mathsf{Bool} \to_1 \mathsf{Bool}$ and $x$ has type $\mathsf{Bool}$ already in typing **let**. Then, $y$'s body $\lambda f.f\ x$ has a monotype $(\mathsf{Bool} \to_\pi r) \to_{\pi'} r$ with no constraints (on multiplicity). There are two generalization results depending on the multiplicity $\pi_y$ of $y$ because the use of $x$ also escapes in the type system.

- If $\pi_y = 1$, the type is generalized into $\forall q\, r. (\mathsf{Bool} \to_\pi r) \to_q r$, where $\pi$ is not generalized because the use of $x$ in $y$'s body is $\pi$.
- If $\pi_y = \omega$, the type is generalized into $\forall p\, q\, r. (\mathsf{Bool} \to_p r) \to_q r$, where $\pi$ is generalized (to $p$) because the use of $x$ in $y$'s body is $\omega$.

A difficulty here is that $\pi_y$ needs to be determined at the definition of $y$, while the constraint on $\pi_y$ is only obtained from the use of $y$.

Our design choice is the latter; the multiplicity of a generalizable **let**-bound variable is $\omega$ in the system. One justification for this choice is that a motivation of polymorphic typing is to enhance reusability, while reuse is not possible for variables with multiplicity 1. Another justification is compatibility with recursive definitions, where recursively-defined variables must have multiplicity $\omega$; it might be confusing, for example, if the multiplicity of a list-manipulation function changes after we change its definition from an explicit recursion to *foldr*.

#### 5.3 Inference Rule for Lets

In summary, the following are our criteria about **let** generalization:

- Only **let**s with polymorphic type annotations are generalized.
- Variables introduced by **let** to be generalized have multiplicity $\omega$.

This idea can be represented by the following typing rule:

$$\frac{\begin{array}{c}\Gamma \Vdash e_1 : \tau_1 \rightsquigarrow \Delta_1; C_1 \quad \{\overline{\pi\alpha}\} = \mathsf{fuv}(\tau_1, C_1) \setminus \mathsf{fuv}(\Gamma) \\ C_1' = \exists\overline{\pi\alpha}.(Q \models^{\tau_1} C_1 \wedge \tau \sim \tau_1) \\ \Gamma\theta_1, x : (\forall \overline{pa}.Q \Rightarrow \tau) \Vdash e_2 : \tau_2 \rightsquigarrow \Delta_2, x^M; C_2\end{array}}{\Gamma \Vdash \textbf{let } x : (\forall \overline{pa}.Q \Rightarrow \tau) = e_1 \textbf{ in } e_2 : \tau_2 \rightsquigarrow \omega\Delta_1 + \Delta_2; C_1' \wedge C_2} \textsc{LetA}$$

(We do not discuss non-generalizable **let** because they are typed as $(\lambda x.e_2)\ e_1$.) Constraints like $\exists\overline{\pi\alpha}.(Q \models^{\tau_1} C_1 \wedge \tau \sim \tau_1)$ above are called *implication constraints* [31], which states that the entailment must hold only by instantiating unification variables in $\overline{\pi\alpha}$. There are two roles of implication constraints. One is to delay the checking because $\tau_1$ and $C_1$ contain some unification variables that will be made concrete after this point by solving $C_2$. The other is to guard constraints; in the above example, since the constraints $C_1 \wedge \tau \sim \tau_1$ hold by assuming $Q$, it is not safe to substitute variables outside $\overline{\pi\alpha}$ in solving the constraints because the equivalence might be a consequence of $Q$; recall that $Q$ affects type equality. We note that there is a slight deviation from the original approach [31]; an implication constraint in our system is annotated by $\tau_1$ to identify for which subset of $\{\overline{\pi\alpha}\}$ the existence of a unique solution is not required and thus quantifier elimination is possible, similarly to Sect. 4.

#### 5.4 Solving Constraints

Now, the set of constraints is extended to include implication constraints.

$$C ::= \bigwedge_i \psi_i \qquad \psi_i ::= \cdots \mid \exists\overline{\pi\alpha}.(Q \models^{\tau} C)$$

As we mentioned above, an implication constraint $\exists\overline{\pi\alpha}.(Q \models^{\tau} C)$ means that $Q \models C$ must hold by substituting $\overline{\pi}$ and $\overline{\alpha}$ with appropriate values, where we do not require uniqueness of solutions for unification variables that do not appear in $\tau$. That is, $Q \Vdash^{\tau}_{\mathrm{simp}} C \rightsquigarrow \top; \theta$ must hold with $\mathsf{dom}(\theta) \subseteq \{\overline{\pi\alpha}\}$.

Then, following OutsideIn(X) [31], we define the *solving judgment* $\overline{\pi\alpha}.Q \Vdash^{\tau}_{\mathrm{solv}} C \rightsquigarrow Q'; \theta$, which states that we solve $(Q, C)$ as $(Q', \theta)$ where $\theta$ only touches variables in $\overline{\pi\alpha}$, where $\tau$ is used for disambiguation (Sect. 4). Let us write $\mathsf{impl}(C)$ for all the implication constraints in $C$, and $\mathsf{simpl}(C)$ for the rest. Then, we can define the inference rules for the judgment simply by recursive simplification, similarly to the original [31].

$$\frac{\begin{array}{c}\overline{\pi\alpha}.\ Q \Vdash^{\tau}_{\mathrm{simpl}} \mathsf{simpl}(C) \rightsquigarrow Q_{\mathrm{r}}; \theta \\ \{\overline{\pi_i\alpha_i}.\ Q \wedge Q_i \wedge Q_{\mathrm{r}} \Vdash^{\tau_i}_{\mathrm{solv}} C_i \rightsquigarrow \top; \theta_i\}_{(\exists\overline{\pi_i\alpha_i}.(Q_i \models^{\tau_i} C_i)) \in \mathsf{impl}(C\theta)}\end{array}}{\overline{\pi\alpha}.\ Q \Vdash^{\tau}_{\mathrm{solv}} C \rightsquigarrow Q_{\mathrm{r}}; \theta}$$

Here, $\overline{\pi\alpha}.\ Q \Vdash^{\tau}_{\mathrm{simpl}} C \rightsquigarrow Q_{\mathrm{r}}; \theta$ is a simplification relation defined similarly to $Q \Vdash^{\tau}_{\mathrm{simp}} C \rightsquigarrow Q_{\mathrm{r}}; \theta$ except that we are allowed to touch only variables in $\overline{\pi\alpha}$. We omit the concrete rules for this version of simplification relation because they are straightforward except that unification caused by S-Uni and S-Eq and quantifier elimination (Sect. 4) are allowed only for variables in $\{\overline{\pi\alpha}\}$.

Accordingly, we also change the typing rules for bindings to use the solving relation instead of the simplification relation.

$$\frac{\begin{array}{ccc}\Gamma \Vdash e : \tau \rightsquigarrow \Delta; C & \mathsf{fuv}(C,\tau).\ \top \Vdash^{\tau}_{\mathrm{solv}} C \rightsquigarrow Q; \theta & \{\overline{\pi\alpha}\} = \mathsf{fuv}(Q, \tau\theta) \\ \overline{p}, \overline{a} : \text{fresh} & \Gamma, f : \forall \overline{pa}.(Q \Rightarrow \tau\theta)[\overline{\alpha \mapsto a, \pi \mapsto p}] \Vdash \mathit{prog} & \end{array}}{\Gamma \Vdash f = e; \mathit{prog}}$$

$$\frac{\Gamma \Vdash e : \sigma \rightsquigarrow \Delta; C \quad \mathsf{fuv}(C,\sigma).\ Q \Vdash^{\sigma}_{\mathrm{solv}} C \wedge \tau \sim \sigma \rightsquigarrow \top; \theta \quad \Gamma, f : \forall \overline{pa}.Q \Rightarrow \tau \Vdash \mathit{prog}}{\Gamma \Vdash f : (\forall \overline{pa}.Q \Rightarrow \tau) = e; \mathit{prog}}$$

Above, there are no unification variables other than $\mathsf{fuv}(C, \tau)$ or $\mathsf{fuv}(C, \sigma)$.

The definition of the solving judgment and the updated inference rules for programs are the same as those in the original OutsideIn(X) [31] except $\tau$ for disambiguation. This is one of the advantages of being based on OutsideIn(X).

## 6 Implementation and Evaluation

In this section, we evaluate the proposed inference method using our prototype implementation. We first report what types are inferred for functions from `Prelude` to see whether or not inferred types are reasonably simple. We then report the performance evaluation that measures efficiency of type inference and the overhead due to entailment checking and quantifier elimination.

### 6.1 Implementation

The implementation follows the present paper except for a few points. Following the implementation of OutsideIn(X) in GHC, our type checker keeps a natural number, which we call an implication level, corresponding to the depth of implication constraints, and a unification variable also accordingly keeps the implication level at which the variable is introduced. As usual, we represent unification variables by mutable references. We perform unification on the fly by destructive assignment, while unification of variables that have smaller implication levels than the current level is recorded for later checking of implication constraints; such a variable cannot be in $\overline{\pi\alpha}$ of $\exists\overline{\pi\alpha}.Q \models^{\tau} C$. The implementation supports GADTs because they can be implemented rather easily by extending constraints $Q$ to include type equalities, but does not support type classes because the handling of them requires another X of OutsideIn(X).

Although we can use a linear-time Horn SAT solving algorithm [10] for checking $Q \models \phi$, the implementation uses a general SAT solver based on DPLL [8, 9] because the unit propagation in DPLL works efficiently for Horn formulas. We do not use external solvers, such as Z3, as we conjecture that the sizes of formulas are usually small, and overhead to use external solvers would be high.

$$
\begin{aligned}
(\circ) &: (q \leq s \wedge q \leq t \wedge p \leq t) \Rightarrow (b \to_q c) \to_r (a \to_p b) \to_s a \to_t c \\
\mathit{curry} &: (p \leq r \wedge p \leq s) \Rightarrow ((a \otimes b) \to_p c) \to_q a \to_r b \to_s c \\
\mathit{uncurry} &: (p \leq s \wedge q \leq s) \Rightarrow (a \to_p b \to_q c) \to_r (a \otimes b) \to_s c \\
\mathit{either} &: (p \leq r \wedge q \leq r) \Rightarrow (a \to_p c) \to_\omega (b \to_q c) \to_\omega \mathsf{Either}\ a\ b \to_r c \\
\mathit{foldr} &: (q \leq r \wedge p \leq s \wedge q \leq s) \Rightarrow (a \to_p b \to_q b) \to_\omega b \to_r \mathsf{List}\ a \to_s b \\
\mathit{foldl} &: (p \leq r \wedge r \leq s \wedge q \leq s) \Rightarrow (b \to_p a \to_q b) \to_\omega b \to_r \mathsf{List}\ a \to_s b \\
\mathit{map} &: (p \leq q) \Rightarrow (a \to_p b) \to_\omega \mathsf{List}\ a \to_q \mathsf{List}\ b \\
\mathit{filter} &: (a \to_p \mathsf{Bool}) \to_\omega \mathsf{List}\ a \to_\omega \mathsf{List}\ a \\
\mathit{append} &: \mathsf{List}\ a \to_p \mathsf{List}\ a \to_q \mathsf{List}\ a \\
\mathit{reverse} &: \mathsf{List}\ a \to_p \mathsf{List}\ a \\
\mathit{concat} &: \mathsf{List}\ (\mathsf{List}\ a) \to_p \mathsf{List}\ a \\
\mathit{concatMap} &: (p \leq q) \Rightarrow (a \to_p \mathsf{List}\ b) \to_\omega \mathsf{List}\ a \to_q \mathsf{List}\ b
\end{aligned}
$$

**Fig. 7.** Inferred types for selected functions from `Prelude` (quantifications are omitted)

#### 6.2 Functions from Prelude

We show how our type inference system works for some polymorphic functions from Haskell's `Prelude`. Since we have not implemented type classes and I/O in our prototype implementation and since we can define copying or discarding functions for concrete first-order datatypes, we focus on the unqualified polymorphic functions. Also, we do not consider the functions that are obviously unrestricted, such as *head* and *scanl*, in this examination. In the implementation of the examined functions, we use natural definitions as possible. For example, a linear-time accumulative definition is used for *reverse*. Some functions can be defined by both explicit recursions and *foldr*/*foldl*; among the examined functions, *map*, *filter*, *concat*, and *concatMap* can be defined by *foldr*, and *reverse* can be defined by *foldl*. For such cases, both versions are tested.

Fig. 7 shows the inferred types for the examined functions. Since the inferred types coincide for the two variations (by explicit recursions or by folds) of *map*, *filter*, *append*, *reverse*, *concat*, and *concatMap*, the results do not refer to these variations. Most of the inferred types look unsurprising, considering the fact that the constraint $p \leq q$ is yielded usually when an input that corresponds to $q$ is used in an argument that corresponds to $p$. For example, consider $\mathit{foldr}\ f\ e\ \mathit{xs}$. The constraint $q \leq r$ comes from the fact that $e$ (corresponding to $r$) is passed as the second argument of $f$ (corresponding to $q$) via a recursive call. The constraint $p \leq s$ comes from the fact that the head of $\mathit{xs}$ (corresponding to $s$) is used as the first argument of $f$ (corresponding to $p$). The constraint $q \leq s$ comes from the fact that the tail of $\mathit{xs}$ is used in the second argument of $f$. A little explanation is needed for the constraint $r \leq s$ in the type of *foldl*, where both $r$ and $s$ are associated with types with the same polarity. Such constraints usually come from recursive definitions. Consider the definition of *foldl*:

$$\mathit{foldl} = \lambda f.\lambda e.\lambda x.\mathbf{case}\ x\ \mathbf{of}\ \{\mathsf{Nil} \to e; \mathsf{Cons}\ a\ y \to \mathit{foldl}\ f\ (f\ e\ a)\ y\}$$

Here, we find that $a$, a component of $x$ (corresponding to $s$), appears in the second argument of *fold* (corresponding to $r$), which yields the constraint $r \leq s$.

Note that the inference results do not contain $\to_1$; recall that there is no problem in using unrestricted inputs linearly, and thus the multiplicity of a linear input can be arbitrary. The results also show that the inference algorithm successfully detected that *append*, *reverse*, and *concat* are linear functions.

It is true that these inferred types indeed leak some internal details into their constraints, but those constraints can be understood only from their extensional behaviors, at least for the examined functions. Thus, we believe that the inferred types are reasonably simple.

#### 6.3 Performance Evaluation

**Table 1.** Experimental results

| Program | LOC | Total Elapsed | SAT Elapsed | SAT (#) | QE Elapsed | QE (#) |
|---|---|---|---|---|---|---|
| `funcs` | 40 | 4.3 | 0.70 | (42) | 0.086 | (15) |
| `gv` | 53 | 3.9 | 0.091 | ( 9) | 0.14 | (17) |
| `app1` | 4 | 0.34 | 0.047 | ( 4) | 0.012 | ( 2) |
| `app10` | 4 | 0.84 | 0.049 | ( 4) | 0.038 | (21) |

(times are measured in ms)

We measured the elapsed time for type checking and the overhead of implication checking and quantifier elimination. The following programs were examined in the experiments: `funcs`: the functions in Fig. 7, `gv`: an implementation of a simple communication in a session-type system GV [17] taken from [18, Section 4] with some modifications,[6] `app1`: a pair of the definitions of *app* and *app*$'$, and `app10`: a pair of the definitions of *app* and $\mathit{app10} = \lambda f.\lambda x.\,\underbrace{\mathit{app}\ \ldots\ \mathit{app}}_{10}\ f\ x$. The former two programs are intended to be miniatures of typical programs. The latter two programs are intended to measure the overhead of quantifier elimination. Although the examined programs are very small, they all involve the ambiguity issues. For example, consider the following fragment of the program `gv`:

```
answer : Int = fork prf calculator $ \c -> left c & \c ->
               send (MkUn 3) c & \c -> send (MkUn 4) c & \c ->
               recv c & \(MkUn z, c) -> wait c & \() -> MkUn z
```

(Here, we used our paper's syntax instead of that of the actual examined code.) Here, both `$` and `&` are operator versions of *app*, where the arguments are flipped in `&`. As well as treatment of multiplicities, the disambiguation is crucial for this expression to have type $\mathsf{Int}$.

The experiments were conducted on a MacBook Pro (13-inch, 2017) with Mac OS 10.14.6, 3.5 GHz Intel Core i7 CPU, and 16 GB memory. GHC 8.6.5 with `-O2` was used for compiling our prototype system.

Table 1 lists the experimental results. Each elapsed time is the average of 1,000 executions for the first two programs, and 10,000 executions for the last two. All columns are self-explanatory except for the # column, which counts the number of

[6] We changed the type of *fork* : $\mathsf{Dual}\ s\ s' \to_\omega (\mathsf{Ch}\ s \to_1 \mathsf{Ch}\ \mathsf{End}) \to_1 (\mathsf{Ch}\ s' \to_1 \mathsf{Un}\ r) \to_1 r$, as their type $\mathsf{Dual}\ s\ s' \Rightarrow (\mathsf{Ch}\ s \to_1 \mathsf{Ch}\ \mathsf{End}) \to_1 \mathsf{Ch}\ s'$ is incorrect for the multiplicity erasing semantics. A minor difference is that we used a GADT to witness duality because our prototype implementation does not support type classes.

executions of corresponding procedures. We note that the current implementation restricts $Q_{\mathrm{w}}$ in S-ENTAIL to be $\top$ and removes redundant constraints afterward. This is why the number of SAT solving in `app1` is four instead of two. For the artificial programs (`app1` and `app10`), the overhead is not significant; typing cost grows faster than SAT/QE costs. In contrast, the results for the latter two show that SAT becomes heavy for higher-order programs (`funcs`), and quantifier elimination becomes heavy for combinator-heavy programs (`gv`), although we believe that the overhead would still be acceptable. We believe that, since we are currently using naive algorithms for both procedures, there is much room to reduce the overhead. For example, if users annotate most general types, the simplification invokes trivial checks $\bigwedge_i \phi_i \models \phi_i$ often. Special treatment for such cases would reduce the overhead.

### 7 Related Work

Borrowing the terminology from Bernardy et al. [7], there are two approaches to linear typing: linearity via arrows and linearity via kinds. The former approaches manage how many times an assumption (i.e., a variable) can be used; for example, in Wadler [33]'s linear $\lambda$ calculus, there are two sort of variables: linear and unrestricted, where the latter variables can only be obtained by decomposing **let** $!x = e_1$ **in** $e_2$. Since primitive sources of assumptions are arrow types, it is natural to annotate them with arguments' multiplicities [7, 12, 22]. For multiplicities, we focused on 1 and $\omega$ following Linear Haskell [6, 7, 26]. Although $\{1, \omega\}$ would already be useful for some domains including reversible computation [19, 35] and quantum computation [2, 25], handling more general multiplicities, such as $\{0, 1, \omega\}$ and arbitrary semirings [12], is an interesting future direction. Our discussions in Sect. 2 and 3, similarly to Linear Haskell [7], could be extended to more general domains with small modifications. In contrast, we rely on the particular domains $\{1, \omega\}$ of multiplicities for the crucial points of our inference, i.e., entailment checking and quantifier elimination. Igarashi and Kobayashi [14]'s linearity analysis for $\pi$ calculus, which assigns input/output usage (multiplicities) to channels, has similarity to linearity via arrows. Multiplicity 0 is important in their analysis to identify input/output only channels. They solve constraints on multiplicities separately in polynomial time, leveraging monotonicity of multiplicity operators with respect to ordering $0 \leq 1 \leq \omega$. Here, $0 \leq 1$ comes from the fact that 1 in their system means "at-most once" instead of "exactly once".

The "linearity via kinds" approaches distinguish types of which values are treated linearly and types of which values are not [21, 24, 28], where the distinction usually is represented by kinds [21, 28]. Interestingly, they also have two function types—function types that belong to the linear kind and those that belong to the unrestricted kind—because the kind of a function type cannot be determined solely by the argument and return types. Mazurak et al. [21] use subkinding to avoid explicit conversions from unrestricted values to linear ones. However, due to the variations of the function types, a function can have multiple incompatible types; e.g., the function *const* can have four incompatible types [24] in the system.

Universal types accompanied by kind abstraction [28] address the issue to some extent; it works well for *const*, but still gives two incomparable types to the function composition ($\circ$) [24]. Morris [24] addresses this issue of principality with qualified typing [15]. Two forms of predicates are considered in the system: $\mathsf{Un}\ \tau$ states that $\tau$ belongs to the unrestricted kind, and $\sigma \leq \tau$ states that $\mathsf{Un}\ \sigma$ implies $\mathsf{Un}\ \tau$. This system is considerably simple compared with the previous systems. Turner et al. [29]'s type-based usage analysis has a similarity to the linearity via kinds; in the system, each type is annotated by usage (a multiplicity) as $(\mathsf{List}\ \mathsf{Int}^{\omega})^{\omega}$. Wansbrough and Peyton Jones [34] extends the system to include polymorphic types and subtyping with respect to multiplicities, and have discussions on multiplicity polymorphism. Mogensen [23] is a similar line of work, which reduces constraint solving on multiplicities to Horn SAT. His system concerns multiplicities $\{0, 1, \omega\}$ with ordering $0 \leq 1 \leq \omega$, and his constraints can involve more operations including additions and multiplications but only in the left-hand side of $\leq$.

Morris [24] uses improving substitutions [16] in generalization, which sometimes are effective for removing ambiguity, though without showing concrete algorithms to find them. In our system, as well as S-Eq, $\mathsf{elim}(\exists \pi.Q)$ can be viewed as a systematic way to find improving substitutions. That is, $\mathsf{elim}(\exists \pi.Q)$ improves $Q$ by substituting $\pi$ with $\min\{M_i \mid \omega \leq M_i \in \Phi_\omega\}$, i.e., the largest possible candidate of $\pi$. Though the largest solution is usually undesirable, especially when the right-hand sides of $\leq$ are all singletons, we can also view that $\mathsf{elim}(\exists \pi.Q)$ substitutes $\pi$ by $\prod_{\mu_i \leq 1 \in \Phi_1} \mu_i$, i.e., the smallest possible candidate.

### 8 Conclusion

We designed a type inference system for a rank 1 fragment of $\lambda^q_{\to}$ [7] that can infer principal types based on the qualified typing system OutsideIn(X) [31]. We observed that naive qualified typing infers ambiguous types often and addressed the issue based on quantifier elimination. The experiments suggested that the proposed inference system infers principal types effectively, and the overhead compared with unrestricted typing is acceptable, though not negligible.

Since we based our work on the inference algorithm used in GHC, the natural expectation is to implement the system into GHC. A technical challenge to achieve this is combining the disambiguation techniques with other sorts of constraints, especially type classes, and arbitrarily ranked polymorphism.

**Acknowledgments**

We thank Meng Wang, Atsushi Igarashi, and the anonymous reviewers of ESOP 2020 for their helpful comments on the preliminary versions of this paper. This work was partially supported by JSPS KAKENHI Grant Numbers 15H02681 and 19K11892, JSPS Bilateral Program, Grant Number JPJSBP120199913, the Kayamori Foundation of Informational Science Advancement, and EPSRC Grant *EXHIBIT: Expressive High-Level Languages for Bidirectional Transformations* (EP/T008911/1).

## References

[1] Aehlig, K., Berger, U., Hofmann, M., Schwichtenberg, H.: An arithmetic for non-size-increasing polynomial-time computation. Theor. Comput. Sci. 318(1-2), 3–27 (2004). 10.1016/j.tcs.2003.10.023

[2] Altenkirch, T., Grattage, J.: A functional quantum programming language. In: 20th IEEE Symposium on Logic in Computer Science (LICS 2005), 26-29 June 2005, Chicago, IL, USA, Proceedings. pp. 249–258. IEEE Computer Society (2005). 10.1109/LICS.2005.1

[3] Baillot, P., Hofmann, M.: Type inference in intuitionistic linear logic. In: Kutsia, T., Schreiner, W., Fernández, M. (eds.) Proceedings of the 12th International ACM SIGPLAN Conference on Principles and Practice of Declarative Programming, July 26-28, 2010, Hagenberg, Austria. pp. 219–230. ACM (2010). 10.1145/1836089.1836118

[4] Baillot, P., Terui, K.: A feasible algorithm for typing in elementary affine logic. In: Urzyczyn, P. (ed.) Typed Lambda Calculi and Applications, 7th International Conference, TLCA 2005, Nara, Japan, April 21-23, 2005, Proceedings. Lecture Notes in Computer Science, vol. 3461, pp. 55–70. Springer (2005). 10.1007/11417170_6

[5] Baillot, P., Terui, K.: Light types for polynomial time computation in lambda calculus. Inf. Comput. **207**(1), 41–62 (2009). 10.1016/j.ic.2008.08.005

[6] Bernardy, J.P., Boespflug, M., Newton, R., Jones, S.P., Spiwack, A.: Linear mini-core. GHC Developpers Wiki, https://gitlab.haskell.org/ghc/ghc/wikis/uploads/ceaedb9ec409555c80ae5a97cc47470e/minicore.pdf, visited Oct. 14, 2019

[7] Bernardy, J., Boespflug, M., Newton, R.R., Peyton Jones, S., Spiwack, A.: Linear haskell: practical linearity in a higher-order polymorphic language. PACMPL **2**(POPL), 5:1–5:29 (2018). 10.1145/3158093

[8] Davis, M., Logemann, G., Loveland, D.W.: A machine program for theorem-proving. Commun. ACM **5**(7), 394–397 (1962). 10.1145/368273.368557

[9] Davis, M., Putnam, H.: A computing procedure for quantification theory. J. ACM **7**(3), 201–215 (1960). 10.1145/321033.321034

[10] Dowling, W.F., Gallier, J.H.: Linear-time algorithms for testing the satisfiability of propositional horn formulae. J. Log. Program. **1**(3), 267–284 (1984). 10.1016/0743-1066(84)90014-1

[11] Gan, E., Tov, J.A., Morrisett, G.: Type classes for lightweight substructural types. In: Alves, S., Cervesato, I. (eds.) Proceedings Third International Workshop on Linearity, LINEARITY 2014, Vienna, Austria, 13th July, 2014. EPTCS, vol. 176, pp. 34–48 (2014). 10.4204/EPTCS.176.4

[12] Ghica, D.R., Smith, A.I.: Bounded linear types in a resource semiring. In: Shao, Z. (ed.) Programming Languages and Systems - 23rd European Symposium on Programming, ESOP 2014, Held as Part of the European Joint Conferences on Theory and Practice of Software, ETAPS 2014, Grenoble, France, April 5-13, 2014, Proceedings. Lecture Notes in Computer Science, vol. 8410, pp. 331–350. Springer (2014). 10.1007/978-3-642-54833-8_18

[13] Girard, J., Scedrov, A., Scott, P.J.: Bounded linear logic: A modular approach to polynomial-time computability. Theor. Comput. Sci. **97**(1), 1–66 (1992). 10.1016/0304-3975(92)90386-T

[14] Igarashi, A., Kobayashi, N.: Type reconstruction for linear -calculus with I/O subtyping. Inf. Comput. **161**(1), 1–44 (2000). 10.1006/inco.2000.2872

[15] Jones, M.P.: Qualified Types: Theory and Practice. Cambridge University Press, New York, NY, USA (1995)

[16] Jones, M.P.: Simplifying and improving qualified types. In: Williams, J. (ed.) Proceedings of the seventh international conference on Functional programming languages and computer architecture, FPCA 1995, La Jolla, California, USA, June 25-28, 1995. pp. 160–169. ACM (1995). 10.1145/224164.224198

[17] Lindley, S., Morris, J.G.: A semantics for propositions as sessions. In: Vitek, J. (ed.) Programming Languages and Systems - 24th European Symposium on Programming, ESOP 2015, Held as Part of the European Joint Conferences on Theory and Practice of Software, ETAPS 2015, London, UK, April 11-18, 2015. Proceedings. Lecture Notes in Computer Science, vol. 9032, pp. 560–584. Springer (2015). 10.1007/978-3-662-46669-8_23

[18] Lindley, S., Morris, J.G.: Embedding session types in haskell. In: Mainland, G. (ed.) Proceedings of the 9th International Symposium on Haskell, Haskell 2016, Nara, Japan, September 22-23, 2016. pp. 133–145. ACM (2016). 10.1145/2976002.2976018

[19] Lutz, C.: Janus: a time-reversible language. *Letter to R. Landauer*. (1986), available on: http://tetsuo.jp/ref/janus.pdf

[20] Matsuda, K.: Modular inference of linear types for multiplicity-annotated arrows (2020), http://arxiv.org/abs/1911.00268v2

[21] Mazurak, K., Zhao, J., Zdancewic, S.: Lightweight linear types in system fdegree. In: TLDI. pp. 77–88. ACM (2010)

[22] McBride, C.: I got plenty o’ nuttin’. In: Lindley, S., McBride, C., Trinder, P.W., Sannella, D. (eds.) A List of Successes That Can Change the World - Essays Dedicated to Philip Wadler on the Occasion of His 60th Birthday. Lecture Notes in Computer Science, vol. 9600, pp. 207–233. Springer (2016). 10.1007/978-3-319-30936-1_12

[23] Mogensen, T.Æ.: Types for 0, 1 or many uses. In: Clack, C., Hammond, K., Davie, A.J.T. (eds.) Implementation of Functional Languages, 9th International Workshop, IFL’97, St. Andrews, Scotland, UK, September 10-12, 1997, Selected Papers. Lecture Notes in Computer Science, vol. 1467, pp. 112–122. Springer (1997). 10.1007/BFb0055427

[24] Morris, J.G.: The best of both worlds: linear functional programming without compromise. In: Garrigue, J., Keller, G., Sumii, E. (eds.) Proceedings of the 21st ACM SIGPLAN International Conference on Functional Programming, ICFP 2016, Nara, Japan, September 18-22, 2016. pp. 448–461. ACM (2016). 10.1145/2951913.2951925

[25] Selinger, P., Valiron, B.: A lambda calculus for quantum computation with classical control. Mathematical Structures in Computer Science **16**(3), 527–552 (2006). 10.1017/S0960129506005238

[26] Spiwack, A., Domínguez, F., Boespflug, M., Bernardy, J.P.: Linear types. GHC Proposals, https://github.com/tweag/ghc-proposals/blob/linear-types2/proposals/0000-linear-types.rst, visited Sep. 11, 2019

[27] Stuckey, P.J., Sulzmann, M.: A theory of overloading. ACM Trans. Program. Lang. Syst. **27**(6), 1216–1269 (2005). 10.1145/1108970.1108974

[28] Tov, J.A., Pucella, R.: Practical affine types. In: Ball, T., Sagiv, M. (eds.) Proceedings of the 38th ACM SIGPLAN-SIGACT Symposium on Principles of Programming Languages, POPL 2011, Austin, TX, USA, January 26-28, 2011. pp. 447–458. ACM (2011). 10.1145/1926385.1926436

[29] Turner, D.N., Wadler, P., Mossin, C.: Once upon a type. In: Williams, J. (ed.) Proceedings of the seventh international conference on Functional programming languages and computer architecture, FPCA 1995, La Jolla, California, USA, June 25-28, 1995. pp. 1–11. ACM (1995). 10.1145/224164.224168

[30] Vytiniotis, D., Peyton Jones, S.L., Schrijvers, T.: Let should not be generalized. In: Kennedy, A., Benton, N. (eds.) Proceedings of TLDI 2010: 2010 ACM SIGPLAN International Workshop on Types in Languages Design and Implementation, Madrid, Spain, January 23, 2010. pp. 39–50. ACM (2010). 10.1145/1708016.1708023

[31] Vytiniotis, D., Peyton Jones, S.L., Schrijvers, T., Sulzmann, M.: Outsidein(x) modular type inference with local assumptions. J. Funct. Program. **21**(4-5), 333–412 (2011). 10.1017/S0956796811000098

[32] Wadler, P.: Linear types can change the world! In: Broy, M. (ed.) Programming concepts and methods: Proceedings of the IFIP Working Group 2.2, 2.3 Working Conference on Programming Concepts and Methods, Sea of Galilee, Israel, 2-5 April, 1990. p. 561. North-Holland (1990)

[33] Wadler, P.: A taste of linear logic. In: Borzyszkowski, A.M., Sokolowski, S. (eds.) Mathematical Foundations of Computer Science 1993, 18th International Symposium, MFCS’93, Gdansk, Poland, August 30 - September 3, 1993, Proceedings. Lecture Notes in Computer Science, vol. 711, pp. 185–210. Springer (1993). 10.1007/3-540-57182-5_12

[34] Wansbrough, K., Peyton Jones, S.L.: Once upon a polymorphic type. In: Appel, A.W., Aiken, A. (eds.) POPL ’99, Proceedings of the 26th ACM SIGPLAN-SIGACT Symposium on Principles of Programming Languages, San Antonio, TX, USA, January 20-22, 1999. pp. 15–28. ACM (1999). 10.1145/292540.292545

[35] Yokoyama, T., Axelsen, H.B., Glück, R.: Towards a reversible functional language. In: Vos, A.D., Wille, R. (eds.) RC. Lecture Notes in Computer Science, vol. 7165, pp. 14–29. Springer (2011). 10.1007/978-3-642-29517-1_2

